# Zinc borylation and reduction by a diborane(4) species *via* B–O bond formation†

**DOI:** 10.1039/d4sc06389a

**Published:** 2024-11-04

**Authors:** Liam P. Griffin, Simon Aldridge

**Affiliations:** a Inorganic Chemistry Laboratory, Department of Chemistry, University of Oxford South Parks Road Oxford OX1 3QR UK simon.aldridge@chem.ox.ac.uk

## Abstract

We report a convenient synthesis of the zinc–boryl complex (Nacnac^Mes^)ZnBpin under mild conditions *via* formal disproportionation of bis(pinacolato)diboron(4), aided thermodynamically by B–O bond formation. This species can be isolated both base-free and as the DMAP adduct (Nacnac^Mes^)Zn(DMAP)Bpin and crystallographically characterised in the latter form. Onward reactivity of the base free zinc boryl complex with CO_2_ occurs reductively, yielding further B–O bonds as well as CO gas. The strength of this driving force can then be harnessed in the reaction between (Nacnac^Mes^)ZnBpin and a Zn–O bonded species (Nacnac^Mes^)ZnOB{(NDippCH)_2_}, which yields the metal–metal bonded dimer [(Nacnac^Mes^)Zn]_2_, the overall formation of which utilizes a diboron(4) species as the stoichiometric reductant of Zn(ii) to Zn(i).

## Abstract 2

Zinc boryl and metal–metal bonded Zn(i) complexes can be accessed from a common Zn(ii) iodide precursor and bis(pinacolaton)diboron(4).

## Graphical abstract

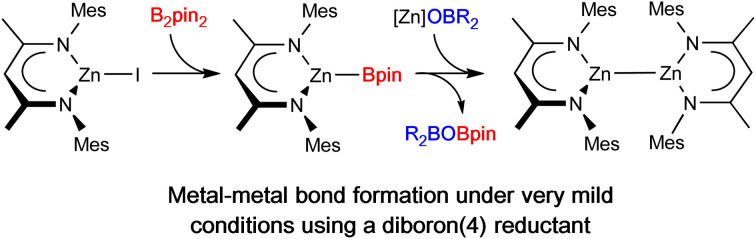

## Introduction

The boryl functional group, BX_2_, is an important motif in organic chemistry, enabling powerful and wide-ranging reactivity, for example in cross-coupling chemistry leading to the formation of C–C and C–X bonds.^1^ Of considerable additional value is the fact that unactivated C–H bonds at both aromatic and aliphatic carbon centres can be borylated, often catalytically, allowing for the introduction of molecular complexity at otherwise hard to activate positions.^2,3^ Such catalytic processes typically proceed *via* metal–boryl complexes generated *in situ*, and in a number of cases the competence of such species as catalytic intermediates has been explicitly demonstrated.^2,4^

Beyond organic synthesis, the boryl anion, [BX_2_]^−^ is also of significant interest in its own right. Featuring a formally anionic B(i) centre, these systems act as X-type ligands, and exert a marked influence on the electronic structure and reactivity of the metal centre to which they are bound, based on their strongly σ-donating and *trans-*labilising properties.^5^ Installation of such ligands is typically achieved by oxidative addition of B–H or B–B bonds at low-valent metal centres.^6^ Alternatively, salt metathesis can be employed, for example using an isolable boryl anion equivalent, an approach facilitated by the landmark synthesis of (THF)_2_Li{B(NDippCH)_2_} (I) by Yamashita, Nozaki *et al.* in 2006.^7–11^ While the latter approach has been utilized in the synthesis of metal boryl complexes from across the Periodic Table, these anions typically require harsh alkali metal reductants, are challenging to store and handle, and (in the case of metathesis at redox-active metals) can lead to side-reactions caused by their strongly reducing nature.^12^ For main group systems (especially divalent complexes), installation of a boryl ligand by oxidative addition is also not trivial, as access to low-valent metal precursors is generally less facile than for their transition metal counterparts.

In seminal work, Hill *et al.* demonstrated that a DMAP-stabilized magnesium boryl reagent (II) could be synthesised by the formal disproportionation of bis(pinacolato)diboron(4), *via* a reaction with an alkylmagnesium reagent.^13^ More broadly, borylation reactions relying on sp^2^-sp^3^ diborane reagents have found widespread application in organic synthesis, with species such as K[^*t*^BuOB_2_pin_2_] (III) allowing the gentle introduction of a (nucleophilic) pinacolato boryl fragment at a carbon electrophile, driven thermodynamically by B–O bond formation and the lattice enthalpy of the potassium halide salt.^14^

**Fig. 1:**
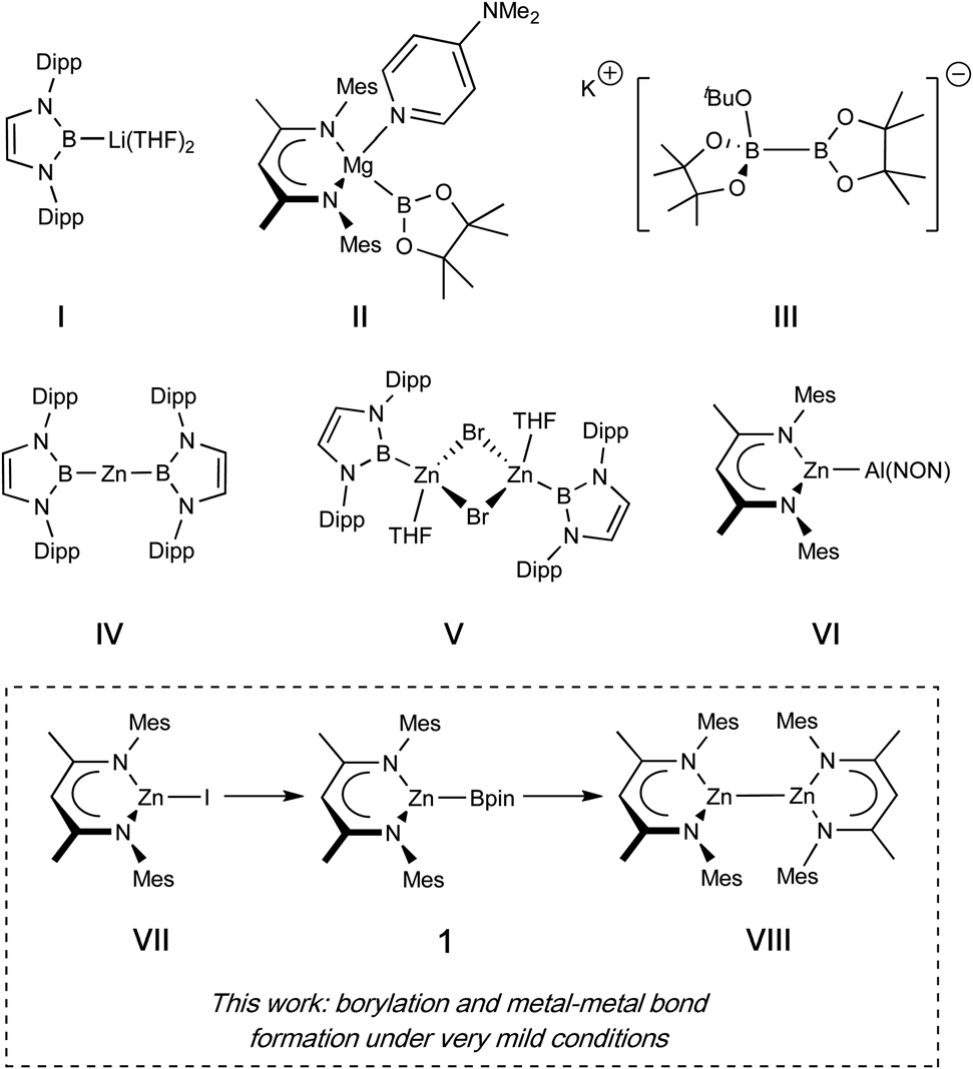
Boryl and related compounds of relevance to the current study.

This report prompted us to investigate whether the introduction of a boryl fragment *via* diborane(4) disproportionation could be accomplished at other main group metals, and whether simple metal halides could serve as alternative precursors, rather than the metal alkyl species used in Hill's studies. In these endeavours we targeted a β-diketiminate (Nacnac) supported zinc boryl complex (1), since such species are relatively rare in the literature (see Fig. 1 for representative examples of zinc boryl complexes, IV/V), and well-characterized examples would permit direct comparison with established magnesium boryl chemistry, and also with heavier zinc aluminyl species (*e.g.*VI). Additionally, we reasoned that the installation of a Zn–B linkage might provide a platform for convenient access to low oxidation state zinc complexes bearing homometallic bonds through the extrusion of B–O bonds in reactions between Zn–B and Zn–OR precursors. Such species (*e.g.*VIII), originating with Carmona's seminal report of decamethyldizincocene, Cp*ZnZnCp*, offer useful insight into fundamental questions of structure and bonding, with multiple examples now known for zinc and magnesium and (most recently) beryllium.^15–17^ Typically, such species are accessed through the use of (very strong) alkali metal reductants, which are often unpredictable and technically challenging. Overall reductive processes in which a more stable, commercially available reductant could be used (*e.g.* the diboron(4) species B_2_pin_2_) could open up a range of ‘soft’ metal–metal bond forming reactions, driven by B–O bond formation. Accordingly, we also report that the combination of zinc boryl and zinc boryloxy complexes leads to elimination of a B–O–B moiety and Zn–Zn bond formation; the preceding formation of both zinc compounds from the same (Nacnac)ZnI precursor (VII), then offers an overall route to Zn(i) from Zn(ii) using a diboron(4) reagent as the net reductant.^4,18–22^

## Results and discussion

### Syntheses of zinc boryl complexes from B_2_pin_2_

**Scheme 1:**
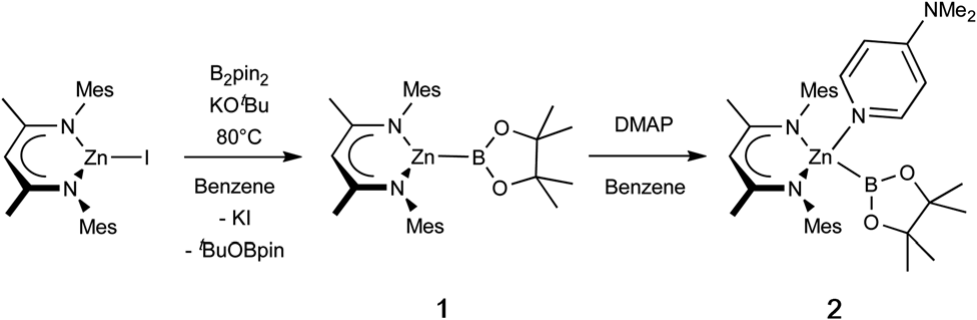
Synthesis of base-free zinc-boryl species (Nacnac^Mes^)ZnBpin (1), and subsequent DMAP coordination.

In targeting a complex of type 1(Scheme 1), the reactions of (THF)_2_Li{B(NDippCH)_2_} (I) with Nacnac-ligated zinc halides were initially trialled, but these yielded the previously reported zinc bis(boryl) species Zn{B(NDippCH)_2_}_2_ (IV) and the lithiated Nacnac ligand, presumably due to the thermodynamic stability of the homoleptic product.^4^ As such, ‘soft’ introduction of the boryl ligand by diborane disproportionation was examined instead. Pre-mixing KO^*t*^Bu and B_2_pin_2_ in benzene, followed by heating to 80 °C for 1 h, with occasional sonication yielded a homogeneous suspension. Subsequent addition of the zinc iodide precursor (Nacnac^Mes^)ZnI (VII),^23^ and heating to 80 °C for 16 h leads to clean and complete conversion of the zinc starting material to one new solution-phase species, as well as one equivalent of ^*t*^BuOBpin (as identified by ^1^H and ^11^B NMR spectroscopy).^24^ The new species gives rise to a relatively broad low-field ^11^B NMR resonance at 38.7 ppm, which remains unchanged between ^1^H coupled and decoupled spectra. The ^1^H NMR spectrum is consistent with a 1 : 1 ratio of pinacolate and Nacnac signals, as well as a symmetrical environment for both fragments, suggesting the formation of zinc boryl complex 1 (Scheme 1). 1 is both highly soluble and highly sensitive, and single crystals suitable for X-ray crystallographic analysis could not be obtained in our hands. Spectroscopic and reactivity data are, however, consistent with its characterisation as (Nacnac^Mes^)ZnBpin. *In situ* generated solutions are stable for several days in a rigorously air- and moisture-free environment (even at temperatures up to 80 °C) and were used for onward reactivity, as discussed below. Interestingly, no reaction was observed under equivalent conditions with the bulkier Dipp-substituted β-diketiminate zinc iodide precursor, (Nacnac^Dipp^)ZnI, presumably on steric grounds.

**Fig. 2:**
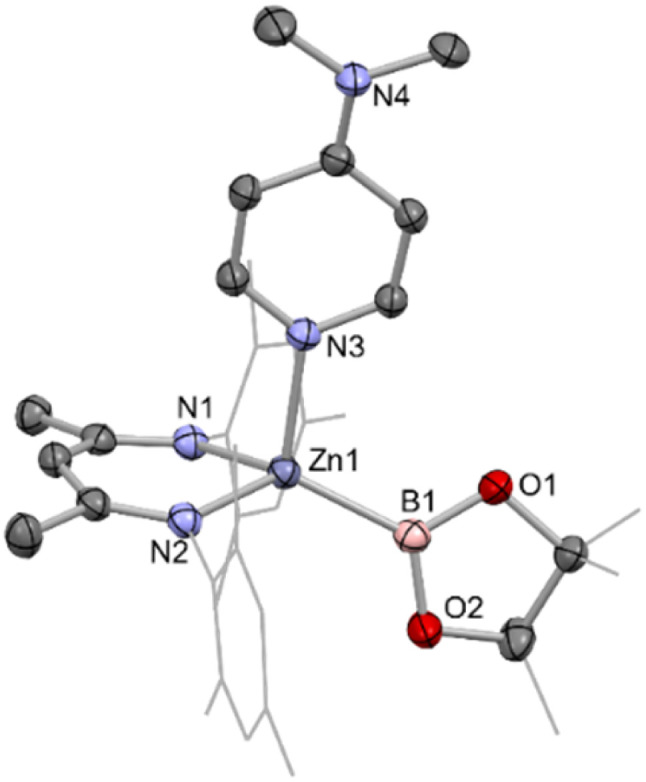
Molecular structure of one of the two crystallographically independent molecules present in the asymmetric unit of (Nacnac^Mes^)Zn(DMAP)Bpin (2), as determined by single crystal X-ray crystallography. Hydrogen atoms omitted and selected residues displayed in wireframe for clarity. Key bond lengths (Å) and bond angles (°): Zn1–B1 2.085(2) Zn1–N1 2.028(1), Zn1–N2 1.025(1), Zn1–N3 2.119(1), B1–Zn1–N3 118.1(7), N1–Zn1–N2 93.86(6).

Addition of the strong donor *N*,*N*-dimethylaminopyridine (DMAP) to an *in situ* generated solution of (Nacnac^Mes^)ZnBpin (1) leads to the formation of a new species as shown by ^1^H NMR monitoring, which features a 1 : 1 : 1 ratio of Nacnac, DMAP and pinacolate resonances, as well as a further downfield shifted (broad) ^11^B NMR resonance at 41.3 ppm (Scheme 1). This adduct (2) is highly crystalline, and single colourless needles could be obtained from hexane. Crystallographic studies show that the pinacolato-boryl fragment had indeed been installed at the zinc centre under relatively mild conditions *via* diborane dispro-portionation (Fig. 2). However, relative to base-free 1, DMAP complex (Nacnac^Mes^)Zn(DMAP)Bpin (2) is thermally labile, showing significant decomposition after a few hours at 80 °C.

The asymmetric unit of 2 features two crystallographically independent molecules; the geometric parameters for each are very similar (and only one is discussed here). The Zn–B bond length (2.085(2) Å) is the longest example featuring a 4-coordinate zinc centre (range: 2.062(2)–2.079(2) Å), with the remaining 2-coordinate species spanning a wider range which encompasses that of 2 (2.052(3)–2.139(2) Å).^4,18–21^ It can also be put into context by comparison with the sum of the respective covalent radii (2.06 Å).^25^ The zinc centre adopts a distorted tetrahedral geometry, closely resembling that of magnesium analogue II;^13^ the Mg–B bond length (while significantly longer) is also in excess of the sum of respective covalent radii (2.324(2), *cf.* 2.26 Å). The most significant structural difference is in the angle between the Bpin and DMAP ligands (∠B1–Zn1–N3 = 111.81(7); ∠B1–Mg1–N3 = 104.50(7) °), likely due to increased steric repulsion at the smaller metal centre.

**Fig. 3:**
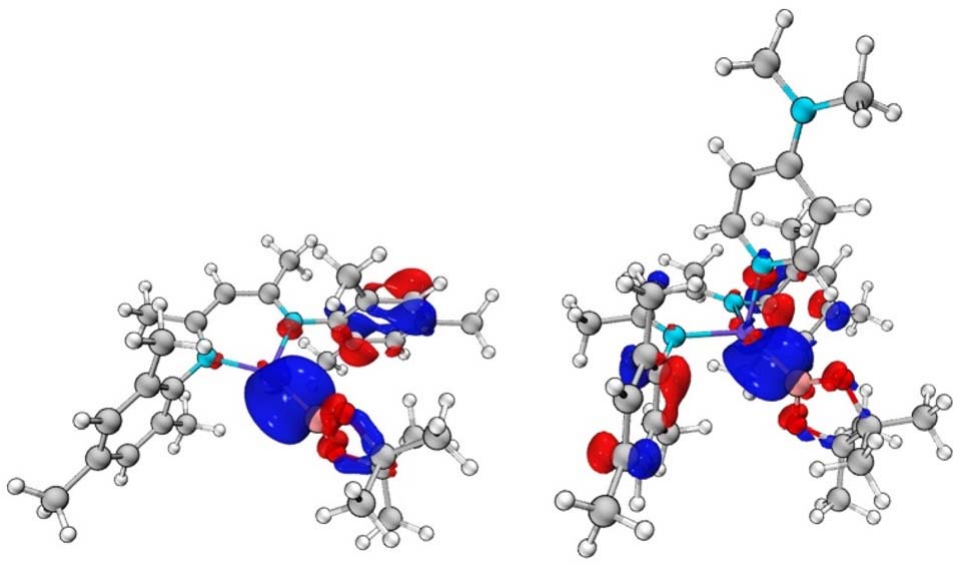
Key molecular orbitals with Zn–B σ-bonding character: (left) HOMO-4 of (Nacnac^Mes^)ZnBpin (1), *E* = −8.92 eV; (right) HOMO-3 of (Nacnac^Mes^)Zn(DMAP)Bpin (2), *E* = −8.24 eV.

DFT calculations were carried out on both the DMAP-complexed and base-free species to probe the nature of the Zn–B interaction in 1 and 2 (see ESI†). In the case of DMAP adduct 2, the HOMO-2 and HOMO-3 contain significant Zn–B sigma bond contributions (Fig. 3), while the LUMO is predominantly localised on the DMAP ring. At 8.57 eV, the HOMO–LUMO gap is very large. The Wiberg Bond Index (WBI) for the Zn–B bond is calculated to be 0.496, consistent with the idea of a weak single bond. In the case of base-free system 1, the HOMO–LUMO gap is larger, at 9.11 eV. However, both the frontier orbitals are ligand based, with no significant contributions to Zn–B bonding. Orbitals of this character are found lower in the manifold, with the HOMO-4 exhibiting the most significant Zn–B σ-bonding character (Fig. 3). The WBI for the Zn–B bond is 0.592, *i.e.* somewhat higher than in the DMAP coordinated case. This is consistent with the fully optimised geometric structures, which feature a shorter Zn–B bond length for 1 (2.041 Å) compared to 2 (2.067 Å).

In order to further probe the Zn–B interactions, natural bond orbital (NBO) analyses were undertaken. A conventional NBO was located for the Zn–B bond only in the case of 1. The bond is strongly polarized towards the boron centre (78.3%), and is almost entirely s based at zinc (96.5%), with a relatively even s/p split at boron (47.6% s, 52.4% p). By contrast, for 2, no NBO was located for the Zn–B interaction. Instead, this interaction is described as a second order perturbation, in which the boron centred lone pair donates into a zinc s-based vacant orbital, with an overall stabilization energy of 232.8 kcal mol^−1^. This difference in the descriptions of the two Zn–B interactions, along with the differing WBIs speaks to a weaker and more labile Zn–B bond in the DMAP coordinated case (presumably as a result of a more crowded zinc centre), resulting in the observed decrease in stability. The more polarized ionic nature in 2 is also borne out in the Bader charges calculated for the two systems, which return a higher positive charge at zinc (0.98, *cf.* 0.88) and a lower charge at boron (1.27, *cf.* 1.35).

Finally, Quantum Theory of Atoms in Molecules (QTAIM) analysis was undertaken to further compare the Zn–B bonding in 1 and 2. In both cases a Bond Critical Point (BCP) could be located along the bond path between zinc and boron. In each case the parameters (a low electron density and small positive Laplacian of electron density) are consistent with ionic bonding. The most significant difference is observed between the two values of the Laplacian, with a more positive value for the DMAP-coordinated species being consistent with a more ionic bond.^26^

### Onward reactivity of zinc boryl complexes 1 and 2 proceeding *via* B–O bond formation

**Scheme 2:**
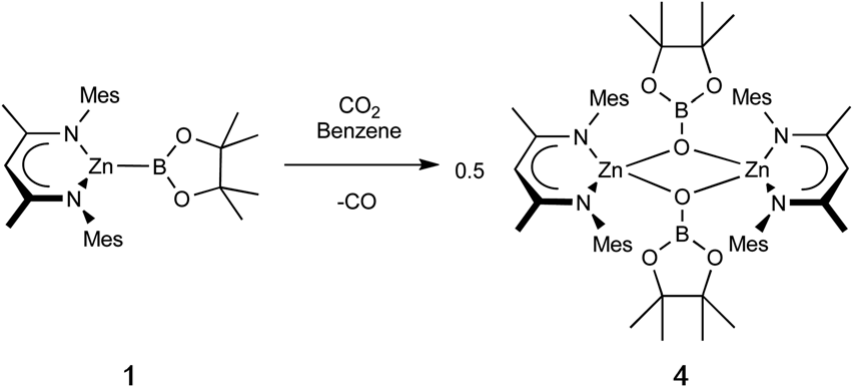
Reaction of 1 with CO_2_ to yield dimeric boryloxy complex 4.

**Fig. 4:**
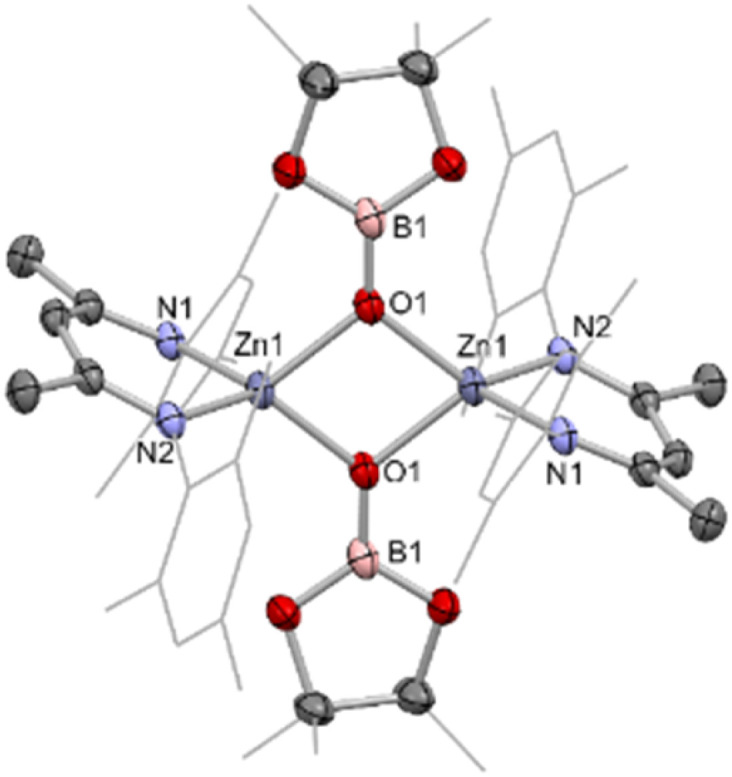
Molecular structure of 4, as determined by single crystal X-ray crystallography. Hydrogen atoms omitted and some residues displayed as wireframe for clarity. Key bond lengths (Å) and bond angles (°): Zn1–O1 1.963(1), Zn1–O1′ 2.012(1), Zn1–N1 1.966(1), Zn1–N2 1.972(1), B1–O1 1.331(2), B1–O2 1.401(8), B2–O3 1.36(1), O1–Zn1–O1′ 80.78(4), N1–Zn1–N1 97.70(5).

Zinc boryl complexes have previously been shown to act as sources of the boryl fragment in (Pd-catalysed) C–B bond forming reactions,^4^ and we expected both 1 and 2 to evidence nucleophilic reactivity at the boryl centre based (simplistically) on the relative electronegativities of Zn (1.65) and B (2.04), and more substantively on the above NBO data.^27^ In both cases, this can be confirmed experimentally by reactivity studies using MeI as a simple electrophilic probe. These yield the known species pinacolato(methyl)borane, and either the zinc iodide starting material (Nacnac^Mes^)ZnI (VII) or its DMAP adduct (Nacnac^Mes^)Zn(DMAP)I (3), which has been characterised crystallographically (Fig. S1†). These results further support our characterisation of (Nacnac^Mes^)ZnBpin.^13,28^ We additionally wished to compare the reactivity of 1 with its heavier analogue Nacnac^Mes^ZnAl(NON) (VI). VI inserts CO_2_ into the metal–metal bond to yield the zinc–carbene complex Nacnac^Mes^ZnCO_2_Al(NON), featuring Zn–C and Al–O bonds, in line with the high oxophilicity of aluminium and the extremely strong electron donating properties of the aluminyl fragment.^22^ Exposure of a solution of 1 to an atmosphere of CO_2_ gas yielded no reaction at room temperature. However, at higher temperatures, conversion to a new species could be seen to occur slowly. Crystallographic analysis reveals that this product is the dimeric boryloxy species [(Nacnac^Mes^)ZnOBpin]_2_ (4), in which each of the assimilated oxygen atoms bridges two zinc and one boron centres (Scheme 2 and Fig. 4). Overall, one molecule of CO_2_ has been reduced to CO per zinc boryl unit. This difference in reactivity between boron and aluminium is likely (at least in part) to be steric in nature, with dimerization presumably facilitated by the small size of the pinacolatoboryloxy fragment.^29–31^

Given the well-known thermodynamic driver associated with the formation of B–O bonds,^32^ we hypothesised that under suitable reaction conditions species 1 might react with a zinc–alkoxide/boryloxide such as 4 to extrude (in this case) O(Bpin)_2_ and yield the metal–metal bonded Zn(i) species [(Nacnac^Mes^)Zn]_2_ (VIII). Intriguingly, close inspection of the ^1^H NMR spectra obtained *in situ* for the reaction between 1 and CO_2_ reveals that VIII is formed in trace quantities. We postulate that it results from a side reaction between as-yet-unreacted zinc boryl complex 1 and the boryloxy product 4 under the relatively harsh reaction conditions employed.^33^

**Fig. 5:**
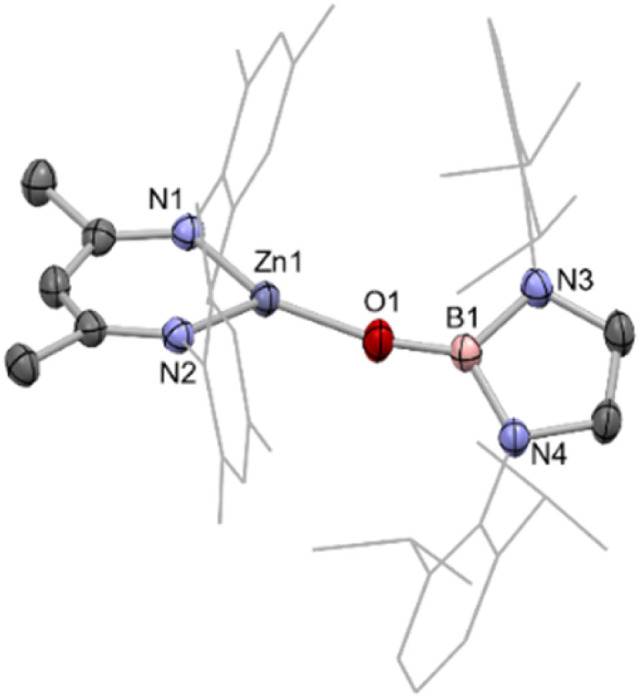
Molecular structure of 5, as determined by X-ray crystallography. Hydrogen atoms omitted and some residues displayed as wireframe for clarity. Key bond lengths (Å) and bond angles (°): Zn1–N1 1.930(1), Zn1–N2 1.942(1), Zn1–O1 1.790(1), B1–O1 1.322(2), B1–N3 1.454(2), B1–N4 1.452(2), N1–Zn1–N2 100.40(5), N1–Zn1–O1 139.57(5), N2–Zn1–O1 119.60(5), Zn1–O1–B1 164.6(1).

Reasoning that this metal–metal bond forming reaction might proceed more cleanly in the case of a monomeric zinc boryloxy species, a complex of this type was targeted, utilizing the bulky N-heterocyclic boryloxy ligand {(HCDippN)_2_}BO–.^34^ Accordingly, the reaction of the protio-ligand {(HCDippN)_2_}BOH with (Nacnac^Mes^)ZnMe cleanly generates the target complex with accompanying release of methane gas.^35^ (Nacnac^Mes^)ZnOB{(NDippCH)_2_} (5) has been characterised by multinuclear NMR and by X-ray crystallography, with the latter revealing a monomeric state of aggregation in the solid state (Fig. 5). NMR-scale reactions between zinc iodide complex VII and the potassiated boryloxy ligand KOB{(NDippCH)_2_} lead to identical product spectra.

**Scheme 3:**
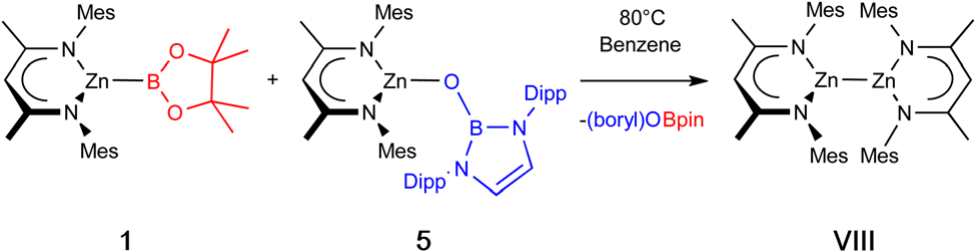
Synthesis of [(Nacnac^Mes^)Zn]_2_ (VIII) *via* metathesis, driven thermodynamically by B–O bond formation.

With 5 in hand, we examined its reaction with 1, which (at 80 °C) can be shown by *in situ* NMR monitoring to yield [(Nacnac^Mes^)Zn]_2_ (VIII) as the major Nacnac-containing species, together with the by-product {(HCDippN)_2_}BOBpin (6), which was characterised spectroscopically (Scheme 3). Further confirmation of the identity of the dizinc product was provided by crystallographic analysis (see ESI†).

Overall, this reaction represents a reduction by bis(pinacolato)diboron(4) of two Zn(ii) centres to two Zn(i) centres in two mild steps which avoid the use of harsh alkali metal reductants. The driving force for each step is the formation of strong B–O bonds. Calculated Gibbs energies indicate that this metal–metal bond forming step is exergonic to the tune of 31.3 kcal mol^−1^. We were further interested to see whether B–O bond formation alone is sufficient to drive the whole process, and equivalent calculations were carried out on the reaction between bis(pinacolato)diboron(4) and two equivalents of (Nacnac^Mes^)ZnOB{(NDippCH)_2_} (5) to yield [(Nacnac^Mes^)Zn]_2_ (VII) and {(HCDippN)_2_}BOBpin (6). These indicate that this process is in principle even more strongly favourable (−41.8 kcal mol^−1^), likely due to the formation of two B–O linkages in a single step. However, experimentally no reaction was observed even at 80 °C, implying that initial installation of the zinc–boryl linkage from 5 and B_2_pin_2_ alone is not kinetically facile.

## Experimental

Included here are synthetic and characterising data for compounds 2, 4 and 5. Data for all compounds, representative ^1^H NMR spectra, and details of crystallographic and computational studies are included in the ESI.† Crystallographic Information Files (CIFs) are available from the Cambridge Crystallographic Data Centre, reference numbers 2385165–2385169.

(Nacnac^Mes^)Zn(DMAP)Bpin (2). To an *in situ* generated solution of (Nacnac^Mes^)ZnBpin (0.1 mmol, 0.7 mL) in C_6_D_6_ (*ca.* 1 mL) was added DMAP (0.012 g, 0.1 mmol). The sample was sonicated briefly to aid dissolution of the DMAP, at which point NMR spectroscopy indicated that conversion was complete. Removal of volatiles *in vacuo*, extraction into hexane and filtration yielded a colourless solution. Single crystals suitable for X-ray crystallography were obtained by slow evaporation of the hexane solution. Yield 0.047 g (73%). ^1^H NMR (500 MHz, benzene-d_6_, 298 K): *δ*_H_ 0.97 (12H, s, {OC(C*H*_3_)_2_}_2_), 1.81 (6H, s, Nacnac-C*H*_3_), 2.13 (6H, s, N(C*H*_3_)_2_), 2.18 (6H, s, *p*-C*H*_3_), 2.27 (12H, s, *o*-C*H*_3_), 5.00 (1H, s, C*H*), 6.02 (2H, d, ^3^*J*_HH_ = 6.24 Hz, DMAP-*m*-C*H*), 6.84 (4H, s, Ar–*H*), 8.64 (2H, d, ^3^*J*_HH_ = 6.24 Hz, DMAP-*o*-C*H*) ppm. ^13^C{^1^H} NMR (101 MHz, benzene-d_6_): *δ*_C_ 19.2 (*o-C*H_3_), 21.0 (*p-C*H_3_), 23.1 (Nacnac-*C*H_3_), 25.7, ({OC(*C*H_3_)_2_}_2_), 38.2 (N(*C*H_3_)_2_), 80.3 (O*C*(CH_3_)_2_), 93.7 (Nacnac-*C*H), 106.6 (DMAP-m-*C*H), 129.2 (Ar-*C*CH_3_), 131.7 (Ar-*C*H), 132.3 (Ar-*C*CH_3_), 147.6 (Ar-*C*N), 150.5 (DMAP-*o-C*H), 154.4 (DMAP-*p-C*N), 165.2 (Nacnac-*C*N) ppm. ^11^B NMR (128 MHz, benzene-d_6_, 298 K): *δ*_B_ 42.2 (br.) ppm. Elemental microanalysis – calculated for C_36_H_51_BN_4_O_2_Zn: C 66.73, H 7.93, N 8.65%. Measured: C 67.04, H 8.00, N 8.27%.

[(Nacnac^Mes^)ZnOBpin]_2_ (4). To a mixture of (Nacnac^Mes^)ZnMe (0.02 g, 0.048 mmol) and HOBpin (0.007 g, 0.049 mmol) was added benzene (0.5 mL), leading to immediate bubbling of the solution, which ceased after *ca.* 1 min. The resulting solution was left to stand, resulting in the formation of large colourless crystals of [(Nacnac^Mes^)ZnOBpin]_2_. The supernatant solution was decanted, and the crystals were washed with hexane (0.5 mL), before removal of all remaining volatiles *in vacuo*. ^1^H NMR (500 MHz, THF-d_8_, 298 K): *δ*_H_ 1.01 (12H, s, {OC(C*H*_3_)_2_}_2_), 1.37 (6H, s, Nacnac-C*H*_3_), 1.78 (12H, s, *o*-C*H*_3_), 2.37 (6H, s, *p*-C*H*_3_), 4.69 (1H, s, C*H*), 6.72 (4H, s, Ar–*H*) ppm. ^13^C{^1^H} NMR (101 MHz, THF-d_8_): *δ*_C_ 18.8 (*o-C*H_3_), 21.4 (*p-C*H_3_), 23.3 (Nacnac-*C*H_3_), 25.4 ({OC(*C*H_3_)_2_}_2_), 81.8 (O*C*(CH_3_)_2_), 95.0 (Nacnac-*C*H), 130.0 (Ar-*C*H), 132.9 (Ar-*C*CH_3_), 133.1 (Ar-*C*CH_3_), 146.5 (Ar-*C*N), 168.1 (Nacnac-*C*N) ppm. ^11^B NMR (128 MHz, THF-d_8_, 298 K): *δ*_B_ 22.6 ppm.

(Nacnac^Mes^)ZnOB{(NDippCH)_2_} (5). To a mixture of (Nacnac^Mes^)ZnMe (0.2 g, 0.48 mmol) and {(HCDippN)_2_}BOH (0.195 g, 0.48 mmol) in a J-Young ampoule was added toluene (5 mL) before stirring at 80 °C for 16 h, during which gas evolution could be observed. Evaporation of volatiles *in vacuo* yielded a sticky solid. Extraction into hexane (5 mL), filtration and removal of solvent *in vacuo* afforded an off-white powder. Single crystals could be obtained by recrystallisation of a portion of this material by slow evaporation from hexane. These crystals were suitable for single crystal X-ray crystallographic measurements. Yield 0.311 g (80%). ^1^H NMR (500 MHz, benzene-d_6_, 298 K): *δ*_H_ 1.00 (12H, d, ^3^*J*_HH_ = 6.91 Hz, CH(C*H*_3_)_2_), 1.21 (12H, d, ^3^*J*_HH_ = 6.91 Hz, CH(C*H*_3_)_2_), 1.36 (6H, s, Nacnac-C*H*_3_), 1.78 (12H, s, *o*-C*H*_3_), 2.24 (6H, s, *p*-C*H*_3_), 3.37 (4H, sept., ^3^*J*_HH_ = 7.01 Hz, C*H*(CH_3_)_2_) 4.73 (1H, s, C*H*), 5.90 (2H, s, {DippNC*H*}_2_) 6.65 (4H, s, Ar–*H*), 7.11 (4H, d (br), ^3^*J*_HH_ = 7.57 Hz, Ar-*m-H*), 7.21 (2H, d (br), ^3^*J*_HH_ = 7.57 Hz, Ar-*p-H*) ppm. ^13^C{^1^H} NMR (101 MHz, benzene-d_6_): *δ*_C_ 18.1 (*o-C*H_3_), 21.1 (*p-C*H_3_), 23.0 (Nacnac-*C*H_3_), 23.7 (CH(*C*H_3_)_2_), 23.9 (CH(*C*H_3_)_2_), 28.7 (*C*H(CH_3_)_2_), 95.6 (Nacnac-*C*H), 115.6 ({DippN*C*H}_2_), 123.3 (Dipp-*m-C*H), 129.9 (Mes-*m-C*H), 131.0 (Mes-*C*CH_3_), 133.9 (Mes-*C*CH_3_), 141.1 (Dipp-*C*N), 143.9 (Mes-*C*N), 146.6 (Dipp-*C*CH(CH_3_)_2_), 170.0 (Nacnac-*C*N) ppm. ^11^B NMR (128 MHz, benzene-d_6_, 298 K): *δ*_B_ 21.5 ppm. Elemental microanalysis – calculated for C_49_H_65_BN_4_OZn: C 73.36, H 8.17, N 6.98%. Measured: C 73.72, H 8.00, N 6.63%.

## Conclusions

In conclusion, we have demonstrated that by exploiting the formation of strong B–O single bonds, a zinc boryl species can be synthesized under mild conditions, and its onward reduction to Zn(i) can also be promoted using the same driving forces, circumventing the need for alkali metal reductants and instead utilizing convenient diboron(4) reagents. In more general terms, this reduction chemistry offers analogies between diboron(4) species and more recently developed Mg–Mg bonded Mg(i) species, in that both can act as mild molecular reductants, albeit with the difference that Mg–Hal *versus* B–O bond-based driving forces mean that their substrate scopes differ significantly (and complementarily).^16^ Work is currently ongoing in our laboratory to extend this chemistry towards the synthesis of new heterobimetallic bonds featuring zinc using B–O bond formation as a thermodynamic driving force.

## Data availability

The data on which this study is based are included in the ESI.† Crystallographic Information Files (CIFs) have been deposited with the Cambridge Crystallographic Data Centre, reference numbers 2385165–2385169. These data can be obtained free of charge from the CCDC *via*https://www.ccdc.cam.ac.uk/data_request/cif.

## Author contributions

LPG: all synthetic experiments, X-ray crystallography, computational work, writing first draft; SA: visualisation, supervision, project administration, funding acquisition, writing, reviewing & editing.

## Conflicts of interest

There are no conflicts to declare.

## Supplementary Material

SC-015-D4SC06389A-s001

SC-015-D4SC06389A-s002
