# Supplementary material for: Zinc borylation and reduction by a diborane(4) species via B–O bond formation
Source: Chem Sci. 2024 Nov 4;15(46):19577–82. doi: 10.1039/d4sc06389a (PMC11575557; doi:10.1039/d4sc06389a)
Supplement: SC-015-D4SC06389A-s001 [file SC-015-D4SC06389A-s001.pdf]

Supplementary Information (SI) for Chemical Science.
This journal is © The Royal Society of Chemistry 2024

Zinc borylation and reduction by a diborane(4) species via B-O bond formation

Liam P. Griffin, Simon Aldridge*

Inorganic Chemistry Laboratory, Department of Chemistry, University of Oxford, South Parks Road, Oxford, OX1 3QR (UK)

Supporting Information (47 pages total)

Table of Contents

1. General Considerations s2
2. X-ray crystallographic details s3
3. Syntheses of novel compounds s6
4. NMR spectra of novel compounds s9
5. Computational details and analysis s26
6. xyz-coordinates of optimised structures s36
7. References for supporting information s47

1. General considerations

All manipulations were carried out using standard Schlenk line or dry-box techniques under an atmosphere of argon or dinitrogen. Solvents were degassed by sparging with argon and dried by passing through a column of the appropriate drying agent. Toluene and hexane were purified using an MBraun SPS-800 solvent purification system and stored over a potassium mirror. THF and Et_2O were purified using an MBraun SPS-800 and stored over activated molecular sieves. NMR spectra were measured in benzene-d_6 (which was dried over potassium, with the solvent then being distilled under reduced pressure), or THF-d_8 (dried by storage over activated molecular sieves and degassed by three freeze pump thaw cycles) and stored under argon in Teflon valve ampoules. NMR samples were prepared under argon in 5 mm Wilmad 507-PP tubes fitted with J. Young Teflon valves. ^{1}H, $^{13}C\{^{1}H\}$, ^{31}P and ^{11}B NMR spectra were recorded on a Bruker Avance III HD nanobay 400 MHz or Bruker Avance III 500 MHz spectrometer at ambient temperature and referenced internally to residual protio-solvent (^{1}H) or solvent (^{13}C) resonances and are reported relative to tetramethylsilane or $Et_2O{\cdot}BF_3$ (δ = 0 ppm). Assignments were confirmed using two-dimensional ^{1}H-^{1}H and ^{13}C-^{1}H NMR correlation experiments. Chemical shifts are quoted in δ (ppm) and coupling constants in Hz. Elemental analyses were carried out by London Metropolitan University. B_2pin_2, HOBpin, DMAP, CO_2 and KO^{t}Bu were used as received. MeI was degassed and stored over molecular sieves. (Nacnac^{Mes})ZnI,[S1] (Nacnac^{Mes})ZnMe,[S2] {(HCDippN)$_2$}BOH[S3] were prepared according to literature procedures.

2. X-ray crystallographic details

Single-crystal X-ray diffraction data for compounds (Nacnac^{Mes})Zn(DMAP)Bpin (**2**), (Nacnac^{Mes})Zn(DMAP)I (**3**), [(Nacnac^{Mes})ZnOBpin]$_2$ (**4**), (Nacnac^{Mes})ZnOB{(NDippCH)$_2$} (**5**) and [(Nacnac^{Mes})Zn]$_2$ (**VII**) were collected on an Oxford Diffraction/Agilent SuperNova diffractometer equipped with a 135 mm Atlas CCD area detector or a Rigaku XtaLAB Synergy-DW VHF equipped with a PhotonJet-R dual wavelength rotating anode and HyPix-Arc 150° detector. Crystals were selected under Paratone-N oil, mounted on MiTeGen Micromount loops and quench-cooled using an Oxford Cryosystems open flow N2 cooling device.[S4] Data were collected at 150 K using mirror monochromated Cu Kα radiation (λ = 1.5418 Å; Oxford Diffraction Supernova) or Mo Kα radiation (λ = 0.71073 Å; Oxford Diffraction Supernova). Data collected were processed using the CrysAlisPro package, including unit cell parameter refinement and inter-frame scaling (which was carried out using SCALE3 ABSPACK within CrysAlisPro).[S5] Equivalent reflections were merged and diffraction patterns processed with the CrysAlisPro suite.[S5] Structures were solved ab initio from the integrated intensities using SHELXT[S6] and refined on F^2 using SHELXL[S7] with the graphical interface OLEX2.[S8] Selected crystallographic data are summarised in Table S1 and full details are given in the supplementary deposited CIF files (CCDC 2385165-2385169). These data can be obtained free of charge from the Cambridge Crystallographic Data Centre via http://www.ccdc.cam.ac.uk/ data_request/cif.

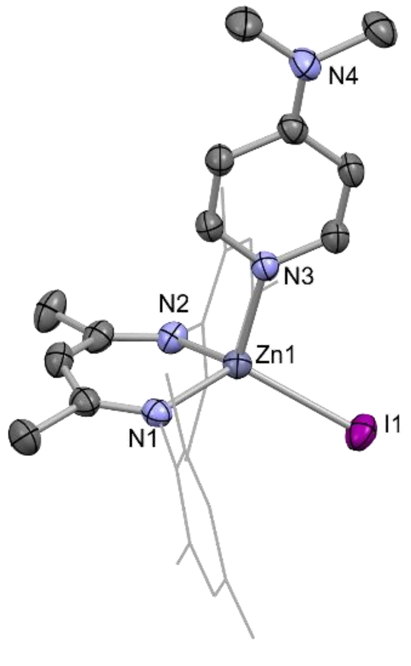

Fig S1. *Molecular structure of (Nacnac^{Mes})Zn(DMAP)I, as determined by single crystal X-ray crystallography. Hydrogen atoms omitted and some residues displayed as wireframe for clarity. Key bond lengths (Å) and bond angles (°): Zn1-N1 2.000(2), Zn1-N2 1.989(2), Zn1-N3 2.047(2), Zn1-I1 2.5572(5), N1-Zn1-N1 96.88(7), N3-Zn1-I1 106.46(5).*

Table S1. *Crystallographic data for compounds (Nacnac^{Mes})Zn(DMAP)Bpin (**2**), (Nacnac^{Mes})Zn(DMAP)I (**3**), [(Nacnac^{Mes})ZnOBpin]$_2$ (**4**), (Nacnac^{Mes})ZnOB{(NDippCH)$_2$} (**5**) and [(Nacnac^{Mes})Zn]$_2$ (**VII**).*

**Compound**	**2**	**3**	**4**
**Formula**	$C_{36}H_{51}BN_4O_2Zn$	$C_{30}H_{39}IN_4Zn(C_6H_{14})$	$C_{58}H_{82}B_2N_4O_6Zn_2$
**Fw (g mol^{-1})**	647.98	734.09	1083.63
**Crystal system**	Monoclinic	Monoclinic	Monoclinic
**Space group**	P 2_1/c	P 2_1/n	P 2_1/n
**a (Å)**	13.88904(6)	8.60930(10)	14.94854(6)
**b (Å)**	26.07802(12)	29.1368(3)	11.80920(5)
**c (Å)**	2016258(9)	14.2475(2)	16.28848(6)
**α (°)**	90	90	90
**β (°)**	92.9187(4)	91.3760(10)	91.3130(4)
**γ (°)**	90	90	90
**V (Å^{-3})**	7293.39(5)	3572.92(7)	2874.654(19)
**Z**	8	4	2
**ρ_{calc} (g cm^{-3})**	1.180	1.365	1.252
**Radiation, λ (Å)**	1.54184	1.54184	1.54184
**Absorption**	Multi-scan	Multi-scan	Multi-scan
**μ (mm^{-1})**	1.196	7.943	1.423
**Reflections collected**	325188	79811	100530
**Independent reflections**	15080	7460	5915
**$R_{(int)}$**	0.0730	0.0466	0.0397
**Parameters**	821	335	414
**R_1 (all data/I>2σ(I))**	0.0386	0.0250	0.0316
**ωR_2 (all data/I >2σ(I))**	0.1124	0.0622	0.0864
**GooF**	1.079	1.039	1.095
**T (K)**	100.00(10)	149.98(16)	100.00(10)
**CCDC reference number**	2385165	2385169	2385167

Table S1 contd.

	5	VIII
**Formula**	$C_{49}H_{65}BN_4OZn$	$C_{46}H_{58}N_4Zn_2$
**Fw (g mol^{-1})**	802.23	797.70
**Crystal system**	Monoclinic	Orthorhombic
**Space group**	P 1 2_1/c 1	P b c a
**a (Å)**	16.1404(2)	21.7767(2)
**b (Å)**	15.15920(10)	10.91670(10)
**c (Å)**	19.0592(2)	36.1028(4)
**α (°)**	90	90
**β (°)**	101.4280(10)	90
**γ (°)**	90	90
**V (Å^{-3})**	4570.87(8)	8582.71(15)
**Z**	4	8
**ρ_{calc} (g cm^{-3})**	1.166	1.235
**Radiation, λ (Å)**	1.54184	1.54184
**Absorption**	Multi-scan	Multi-scan
**μ (mm^{-1})**	1.030	1.626
**Reflections collected**	103585	130609
**Independent reflections**	9545	8834
**$R_{(int)}$**	0.0488	0.0747
**Parameters**	542	485
**R_1 (all data/I>2σ(I))**	0.0319	0.0803
**ωR_2 (all data/I >2σ(I))**	0.0921	0.1829
**GooF**	1.033	1.162
**T (K)**	149.98(11)	100.00(10)
**CCDC reference number**	2385168	2385166

3. Synthesis of novel compounds

(Nacnac^{Mes})ZnBpin (1)

To an NMR tube fitted with a J-Youngs valve was added KO^{t}Bu (0.004 g, 0.04 mmol) and B_2pin_2 (0.01 g, 0.04 mmol) and C_6D_6 (0.5 ml). The suspension was heated to 80 °C for 1 h with occasional ultrasonication to yield a homogeneous mixture. To this was added (Nacnac^{Mes})ZnI (0.02 g, 0.038 mmol) before further heating at 80 °C for 12 h. The resulting solution was filtered to remove precipitated KI and the product was characterised by multinuclear NMR spectroscopy. This indicated quantitative conversion. Also present were signals corresponding to the by-product ^{t}BuOBpin.[S9]

(Nacnac^{Mes})ZnBpin

^{1}H NMR (500 MHz, benzene-d$_6$, 298 K): δ_H 1.02 (12 H, s, {OC(C*H*$_3$)$_2$}$_2$), 1.64 (6 H, s, Nacnac-C*H*$_3$), 2.17 (6 H, s, *p*-C*H*$_3$), 2.20 (12 H, s, *o*-C*H*$_3$), 4.99 (1 H, s, C*H*), 6.82 (4 H, s, Ar-*H*) ppm.

^{13}C{^{1}H} NMR (101 MHz, benzene-d$_6$): δ_C 19.0 (*o*-CH$_3$), 21.0 (*p*-CH$_3$), 22.6 (Nacnac-*C*H$_3$), 25.2, ({OC(*C*H$_3$)-$_2$}$_2$), 83.1 (O*C*(CH$_3$)$_2$), 96.1 (Nacnac-*C*H), 129.3 (Ar-*C*H), 131.2 (Ar-*C*CH$_3$), 133.4 (Ar-*C*CH$_3$), 146.1 (Ar-*C*N), 166.4 (Nacnac-*C*N) ppm.

^{11}B NMR (128 MHz, benzene-d$_6$, 298 K): δ_B 38.8 (br) ppm

^{t}BuOBpin

^{1}H NMR (500 MHz, benzene-d$_6$, 298 K): δ_H 1.06 (12 H, s, {OC(C*H*$_3$)$_2$}$_2$), 1.38 (9 H, s, C(C*H*$_3$)$_3$) ppm.

^{13}C{^{1}H} NMR (101 MHz, benzene-d$_6$): δ_C 24.6 ({OC(*C*H$_3$)$_2$}$_2$), 30.3 (C(*C*H$_3$)$_3$, 73.5 (*C*(CH$_3$)$_3$), 81.76 (O*C*(CH$_3$)$_2$) ppm.

^{11}B NMR (128 MHz, benzene-d$_6$, 298 K): δ_B 21.7 ppm

(Nacnac^{Mes})Zn(DMAP)Bpin (2)

To an *in situ* generated solution of (Nacnac^{Mes})ZnBpin (0.1 mmol, 0.7 mL) in C_6D_6 (ca. 1 mL) was added DMAP (0.012 g, 0.1 mmol). The sample was sonicated briefly to aid dissolution of the DMAP, at which point NMR spectroscopy indicated that conversion was complete. Removal of volatiles *in vacuo*, extraction into hexane and filtration yielded a colourless solution. Single crystals suitable for X-ray crystallography were obtained by slow evaporation of the hexane solution. Yield 0.047 g (73 %).

^{1}H NMR (500 MHz, benzene-d$_6$, 298 K): δ_H 0.97 (12 H, s, {OC(C*H*$_3$)$_2$}$_2$), 1.81 (6 H, s, Nacnac-C*H*$_3$), 2.13 (6 H, s, N(C*H*$_3$)$_2$), 2.18 (6 H, s, *p*-C*H*$_3$), 2.27 (12 H, s, *o*-C*H*$_3$), 5.00 (1 H, s, C*H*), 6.02 (2 H, d, $^3J_{HH}$ = 6.24 Hz, DMAP-*m*-C*H*), 6.84 (4 H, s, Ar-*H*), 8.64 (2 H, d, $^3J_{HH}$ = 6.24 Hz, DMAP-*o*-C*H)* ppm.

^{13}C{^{1}H} NMR (101 MHz, benzene-d$_6$): δ_C 19.2 (*o*-CH$_3$), 21.0 (*p*-CH$_3$), 23.1 (Nacnac-*C*H$_3$), 25.7, ({OC(*C*H$_3$)-$_2$}$_2$), 38.2 (N(*C*H$_3$)$_2$), 80.3 (O*C*(CH$_3$)$_2$), 93.7 (Nacnac-*C*H), 106.6 (DMAP-m-*C*H), 129.2 (Ar-*C*CH$_3$), 131.7 (Ar-*C*H), 132.3 (Ar-*C*CH$_3$), 147.6 (Ar-*C*N), 150.5 (DMAP-*o*-*C*H), 154.4 (DMAP-*p*-*C*N), 165.2 (Nacnac-*C*N) ppm.

^{11}B NMR (128 MHz, benzene-d$_6$, 298 K): δ_B 40.1 (br.) ppm

Elemental Microanalysis – Expected for $C_{36}H_{51}BN_4O_2Zn$: C 66.73, H 7.93, N 8.65 %. Found: C 67.04, H 8.00, N 8.27 %.

Reaction of (Nacnac^{Mes})ZnBpin (1) with MeI

To an *in situ* generated C_6D_6 solution of **1** (0.038 mmol, 0.5 mL) was added MeI (0.02 mL, excess). Immediate reaction yielded the known species (Nacnac^{Mes})ZnI and MeBpin, as determined by *in situ* NMR spectroscopy.

Reaction of (Nacnac^{Mes})Zn(DMAP)Bpin (2) with MeI

To a solution of **2** (0.01 g, 0.015 mmol) in C_6D_6 (0.5 mL) was added MeI (0.02 mL, excess). Immediate reaction yielded the known species MeBpin, as well as a new species. Removal of volatiles, extraction into hexane and filtration yielded a colourless solution, from which colourless single crystals could be obtained when left to stand. Single crystal crystallographic measurements showed that this new species was (Nacnac^{Mes})Zn(DMAP)I, for which an alternative synthesis was conducted by analogy to the reported chloride analogue, yielding a clean sample for the measurement of characterising data.[S10]

(Nacnac^{Mes})Zn(DMAP)I

^{1}H NMR (500 MHz, benzene-d$_6$, 298 K): δ_H 1.63 (6 H, s (br.), *o*-C*H*$_3$), 1.65 (6 H, s, Nacnac-C*H*$_3$), 2.18 (6 H, s, *p*-C*H*$_3$), 2.21 (6 H, s (br.), *o*-C*H*$_3$), 3.05 (6 H, s, N(C*H*$_3$)$_2$), 4.84 (1 H, s, C*H*), 6.66 (2 H, s, Ar-*H*), 6.67 (2 H, br., DMAP-*m*-C*H*), 6.79 (2 H, s, Ar-*H*), 8.20 (2 H, d (br.), $^3J_{HH}$ = 4.86 Hz, DMAP-*o*-C*H)* ppm.

^{13}C{^{1}H} NMR (101 MHz, benzene-d$_6$): δ_C 18.9, 20.1 (*o*-*C*H$_3$), 21.1 (*p*-*C*H$_3$), 23.8 (Nacnac-*C*H$_3$), 39.2 (N(*C*H$_3$)$_2$), 93.9 (Nacnac-*C*H), 107.5 (DMAP-*m*-*C*H), 129.4, 130.0 (Ar-*C*H), 132.0, 133.1, 133.7 (Ar-*C*CH$_3$), 146.3 (Ar-*C*N), 149.6 (DMAP-*o*-*C*H), 156.6 (DMAP-*p*-*C*N), 167.9 (Nacnac-*C*N) ppm.

Reaction of (Nacnac^{Mes})ZnBpin (1) with CO_2

An *in situ* generated C_6D_6 solution of **1** (0.5 mmol, 0.6 mL) was degassed by three freeze-pump-thaw cycles before the addition of one atmosphere of CO_2. Heating to 80 °C for 16 h led to the formation of one predominant species by *in situ* ^{1}H NMR spectroscopy (and CO by ^{13}C{^{1}H} NMR). Removal of volatiles, extraction into hexane and filtration yielded a colourless solution, from which colourless single crystals could be obtained when left to stand. These crystals were suitable for X-ray crystallographic measurements. The minor species observed by NMR exactly matches the signals of the known species [(Nacnac^{Mes})Zn]$_2$. An alternative direct synthesis of [(Nacnac^{Mes})ZnOBpin]$_2$ (**4**) is given below.

[(Nacnac^{Mes})ZnOBpin]$_2$ (**4**)

To a mixture of (Nacnac^{Mes})ZnMe (0.02 g, 0.048 mmol) and HOBpin (0.007 g, 0.049 mmol) was added benzene (0.5 mL), leading to immediate bubbling of the solution, which ceased after ca. 1 min. The resulting solution was left to stand, resulting in the formation of large colourless crystals of [(Nacnac^{Mes})ZnOBpin]$_2$. The supernatant solution was decanted, and the crystals were washed with hexane (0.5 mL), before removal of all remaining volatiles *in vacuo*.

^{1}H NMR (500 MHz, THF-d$_8$, 298 K): δ_H 1.01 (12 H, s, {OC(C*H*$_3$)$_2$}$_2$), 1.37 (6 H, s, Nacnac-C*H*$_3$), 1.78 (12 H, s, *o*-C*H*$_3$), 2.37 (6 H, s, *p*-C*H*$_3$), 4.69 (1 H, s, C*H*), 6.72 (4 H, s, Ar-*H*) ppm.

$^{13}C\{^1H\}$ NMR (101 MHz, THF-d$_8$): δ_C 18.8 (*o*-CH_3), 21.4 (*p*-CH_3), 23.3 (Nacnac-CH_3), 25.4 ($\{OC(CH_3)_2\}_2$), 81.8 ($OC(CH_3)_2$), 95.0 (Nacnac-*C*H), 130.0 (Ar-*C*H), 132.9 (Ar-*C*CH_3), 133.1 (Ar-*C*CH_3), 146.5 (Ar-*C*N), 168.1 (Nacnac-*C*N) ppm.

^{11}B NMR (128 MHz, THF-d$_8$, 298 K): δ_B 22.6 (br.) ppm

(Nacnac^{Mes})ZnOB{(NDippCH)$_2$} (5)

To a mixture of (Nacnac^{Mes})ZnMe (0.2 g, 0.48 mmol) and $\{(HCDippN)_2\}$BOH (0.195 g, 0.48 mmol) in a J-Young ampoule was added toluene (5 mL) before stirring at 80 °C for 16 h, during which gas evolution could be observed. Evaporation of volatiles *in vacuo* yielded a sticky solid. Extraction into hexane (5 mL), filtration and removal of solvent *in vacuo* afforded an off-white powder. Single crystals could be obtained by recrystallisation of a portion of this material by slow evaporation from hexane. These crystals were suitable for single crystal X-ray crystallographic measurements. Yield 0.311 g (80 %).

^1H NMR (500 MHz, benzene-d$_6$, 298 K): δ_H 1.00 (12 H, d, $^3J_{HH}$ = 6.91 Hz, $CH(CH_3)_2$), 1.21 (12 H, d, $^3J_{HH}$ = 6.91 Hz, $CH(CH_3)_2$), 1.36 (6 H, s, Nacnac-CH_3), 1.78 (12 H, s, *o*-CH_3), 2.24 (6 H, s, *p*-CH_3), 3.37 (4 H, sept., $^3J_{HH}$ = 7.01 Hz, $CH(CH_3)_2$) 4.73 (1 H, s, C*H*), 5.90 (2 H, s, $\{DippNCH\}_2$) 6.65 (4 H, s, Ar-*H*), 7.11 (4 H, d (br.), $^3J_{HH}$ = 7.57 Hz, Ar-*m-H*), 7.21 (2 H, d (br.), $^3J_{HH}$ = 7.57 Hz, Ar-*p-H*) ppm.

$^{13}C\{^1H\}$ NMR (101 MHz, benzene-d$_6$): δ_C 18.1 (*o*-CH_3), 21.1 (*p*-CH_3), 23.0 (Nacnac-CH_3), 23.7 ($CH(CH_3)_2$), 23.9 ($CH(CH_3)_2$), 28.7 ($CH(CH_3)_2$), 95.6 (Nacnac-*C*H), 115.6 ($\{DippNCH\}_2$), 123.3 (Dipp-*m*-*C*H), 129.9 (Mes-*m*-*C*H), 131.0 (Mes-*C*CH_3), 133.9 (Mes-*C*CH_3), 141.1 (Dipp-*C*N), 143.9 (Mes-*C*N), 146.6 (Dipp-*C*$CH(CH_3)_2$), 170.0 (Nacnac-*C*N) ppm.

^{11}B NMR (128 MHz, benzene-d$_6$, 298 K): δ_B 21.5 ppm

Elemental Microanalysis – Expected for $C_{49}H_{65}BN_4OZn$: C 73.36, H 8.17, N 6.98 %. Found: C 73.72, H 8.00, N 6.63 %.

Reaction of (Nacnac^{Mes})ZnBpin (1) with (Nacnac^{Mes})ZnOB{(NDippCH)$_2$} (5)

To an *in situ* generated C_6D_6 solution of **1** (0.38 mmol, 0.5 mL) was added **5** (0.03 g, 0.037 mmol), before heating to 80 °C for 12 h. NMR spectroscopy indicates clean conversion to the known species [(Nacnac^{Mes})Zn]$_2$ and one new species, which gives rise to signals consistent with the proposed complex $\{(HCDippN)_2\}$BOBpin.[1] Attempts at crystallisation yielded only crystals of [(Nacnac^{Mes})Zn]$_2$, as well as a stickly gel-like substance assigned to the $\{(HCNDipp)_2\}$BOBpin by-product.

$\{(HCDippN)_2\}$BOBpin

^1H NMR (500 MHz, benzene-d$_6$, 298 K): δ_H 0.69 (12 H, s, $\{OCCH_3\}_2$), 1.25 (12 H, d, $^3J_{HH}$ = 7.00 Hz, $CH(CH_3)_2$), 1.39 (12 H, d, $^3J_{HH}$ = 7.00 Hz, $CH(CH_3)_2$), 3.40 (4 H, sept., $^3J_{HH}$ = 7.00 Hz, $CH(CH_3)_2$), 6.01 (2 H, s, $\{DippNCH\}_2$), 7.15 (4 H, br., Ar-*m-H*), 7.20 (2 H, m, Ar-*p-H*) ppm.

$^{13}C\{^1H\}$ NMR (101 MHz, benzene-d$_6$): δ_C 24.3 ($\{OC(CH_3)_2\}_2$), 24.6 ($CH(CH_3)_2$), 24.8 ($CH(CH_3)_2$), 30.3 ($CH(CH_3)_2$), 82.2 ($OC(CH_3)_2$), 117.0 ($\{DippNCH\}_2$), 123.7, 129.3, 138.0 (Ar-*C*), 146.9 (Ar-*C*N) ppm.

^{11}B NMR (128 MHz, benzene-d$_6$, 298 K): δ_B 20.8, 21.7 ppm

4. NMR Spectra of novel compounds

(Nacnac^{Mes})ZnBpin (1)

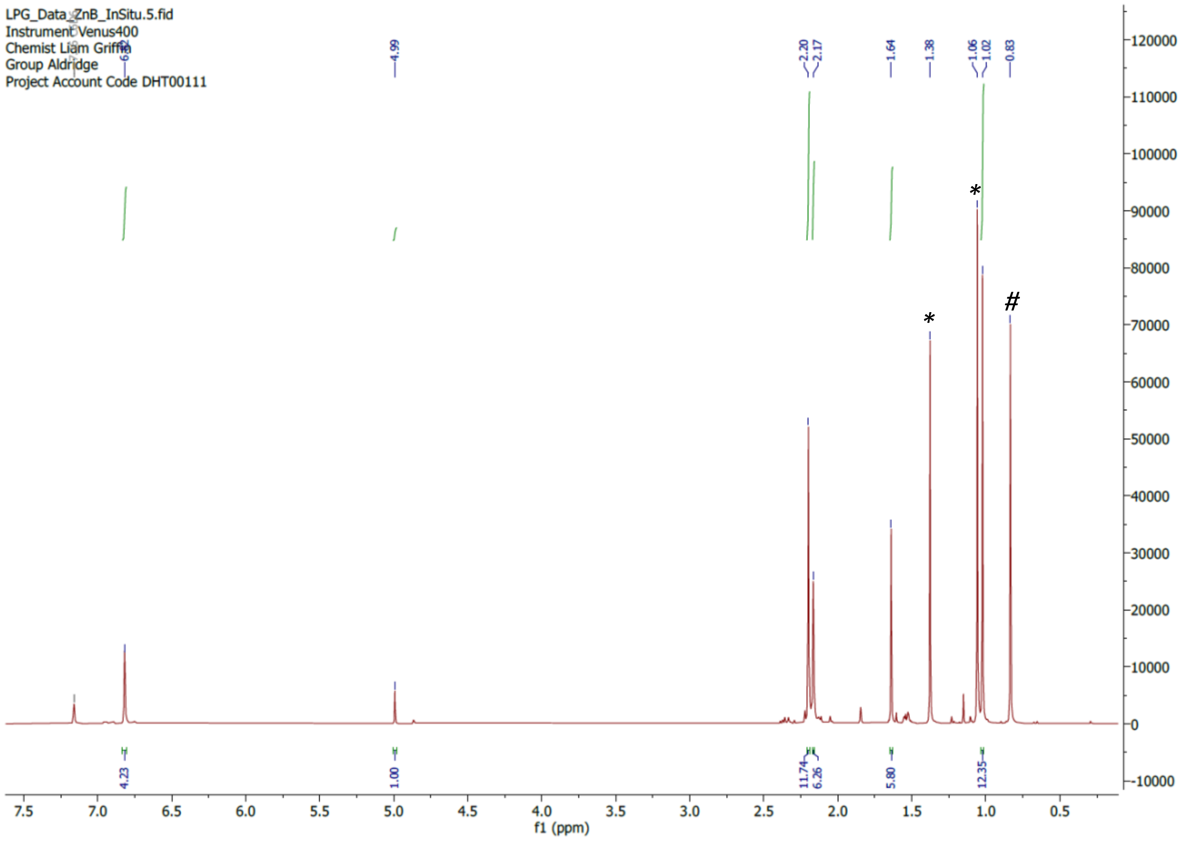

Fig S2. *In situ ^{1}H NMR spectrum of (Nacnac^{Mes})ZnBpin (**1**). ^{t}BuOBPin by-product marked * and residual B_2pin_2 marked #.*

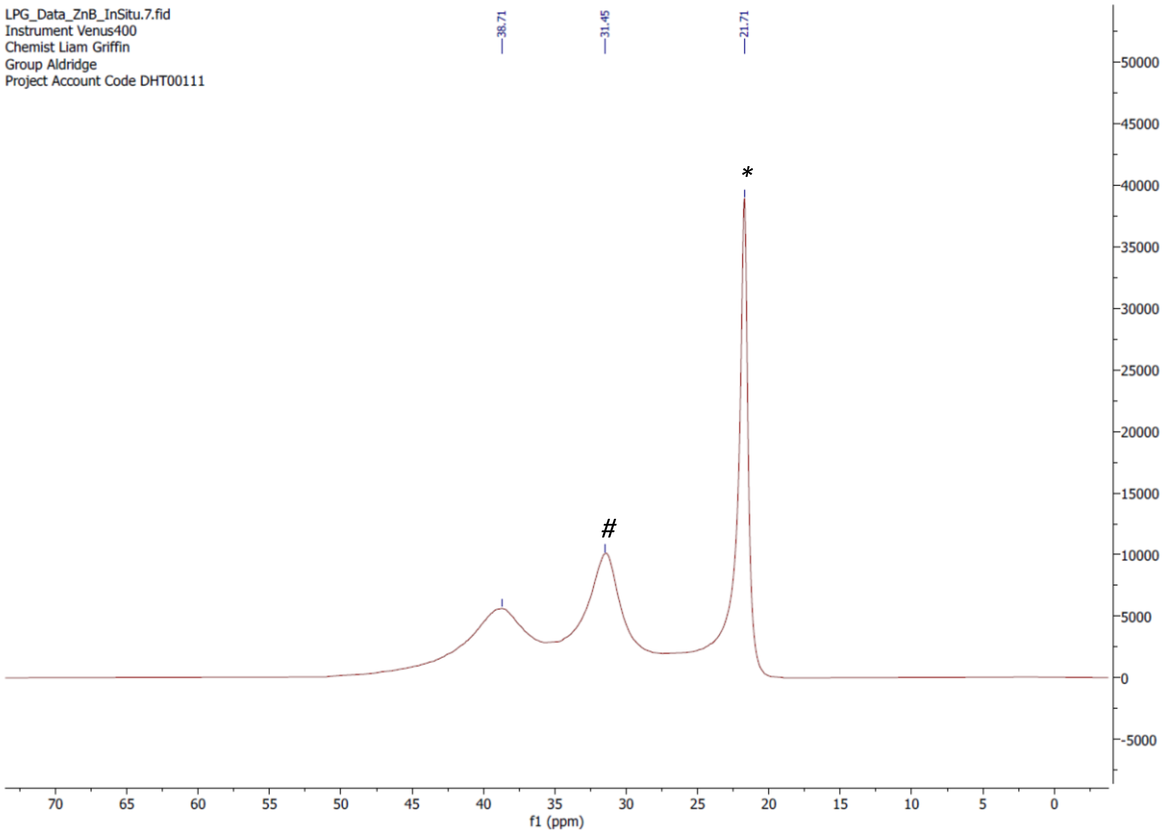

Fig S3. *In situ* ^{11}*B NMR spectrum of (Nacnac*^{Mes}*)ZnBpin (***1***).* ^{t}*BuOBPin by-product marked * and residual* B_2pin_2 *marked #.*

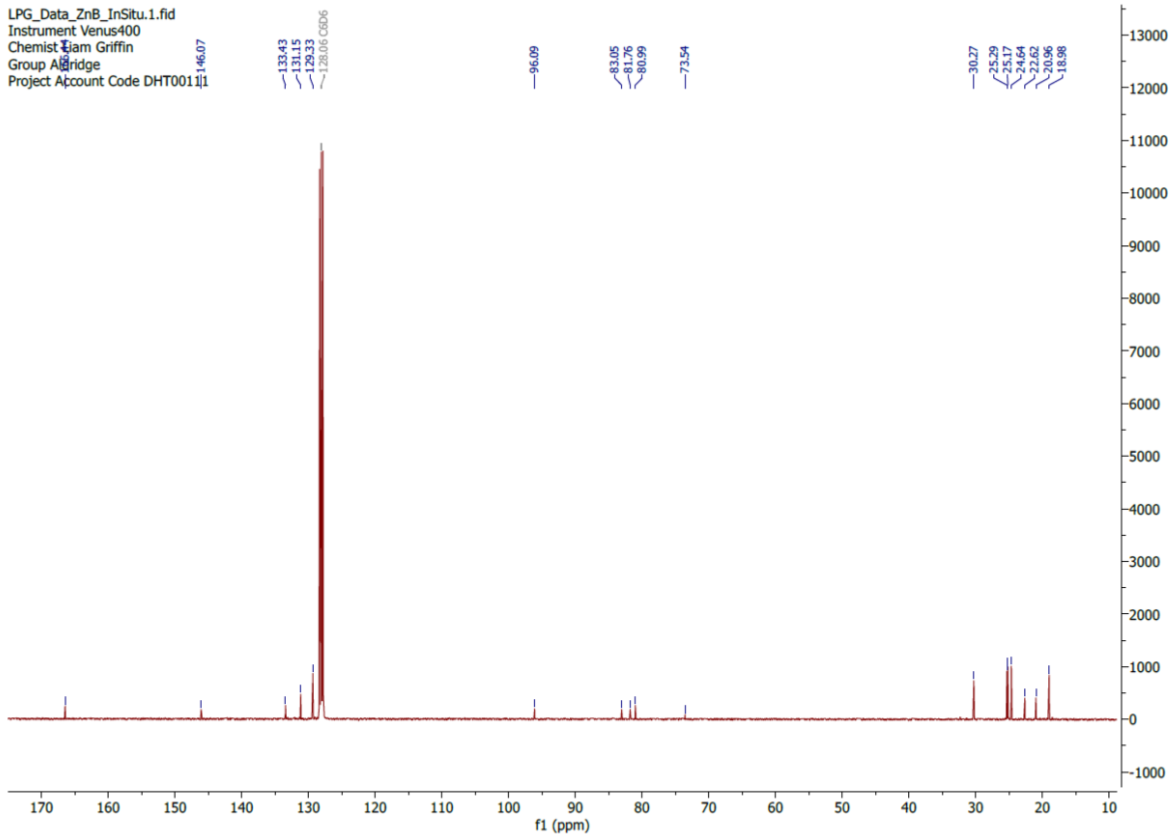

Fig S4. *In situ ^{13}C NMR spectrum of (Nacnac^{Mes})ZnBpin (**1**).*

(Nacnac^{Mes})Zn(DMAP)Bpin (2)

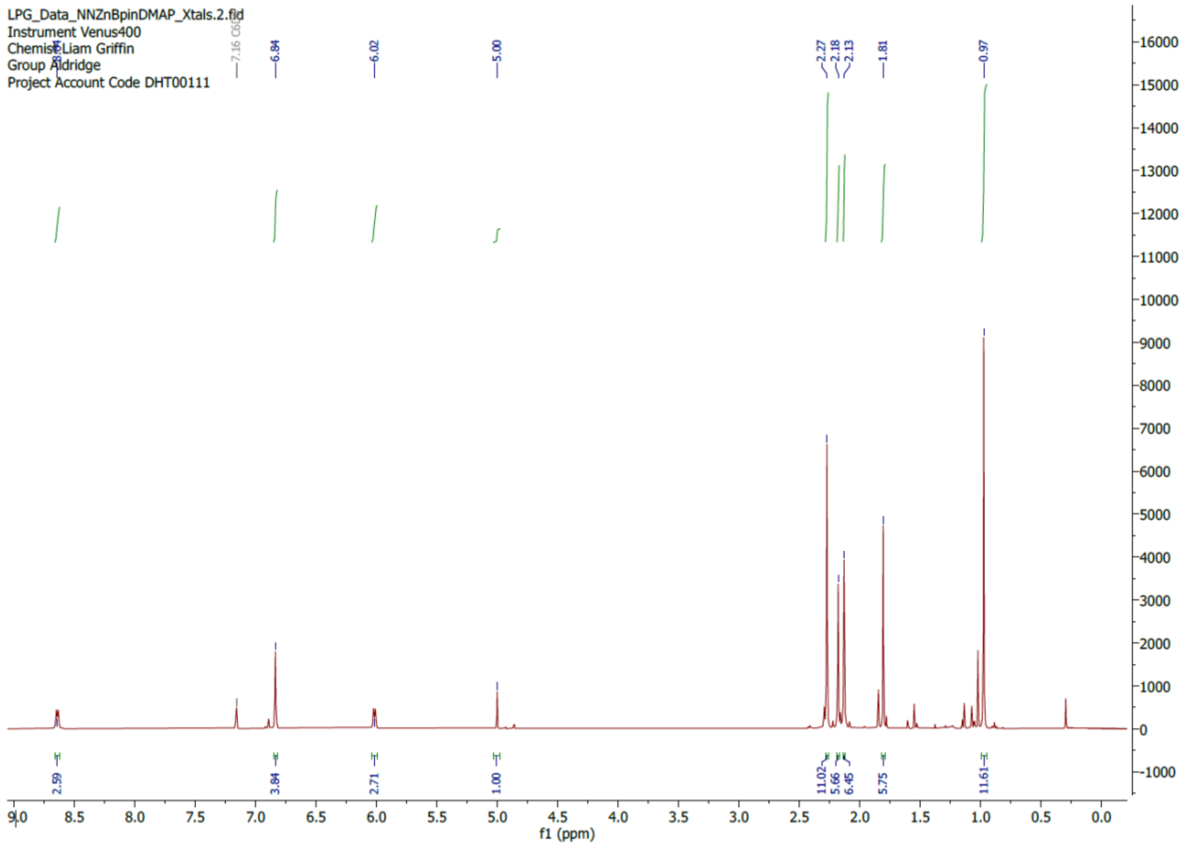

Fig S5. *^{1}H NMR spectrum of (Nacnac^{Mes})Zn(DMAP)Bpin (**2**).*

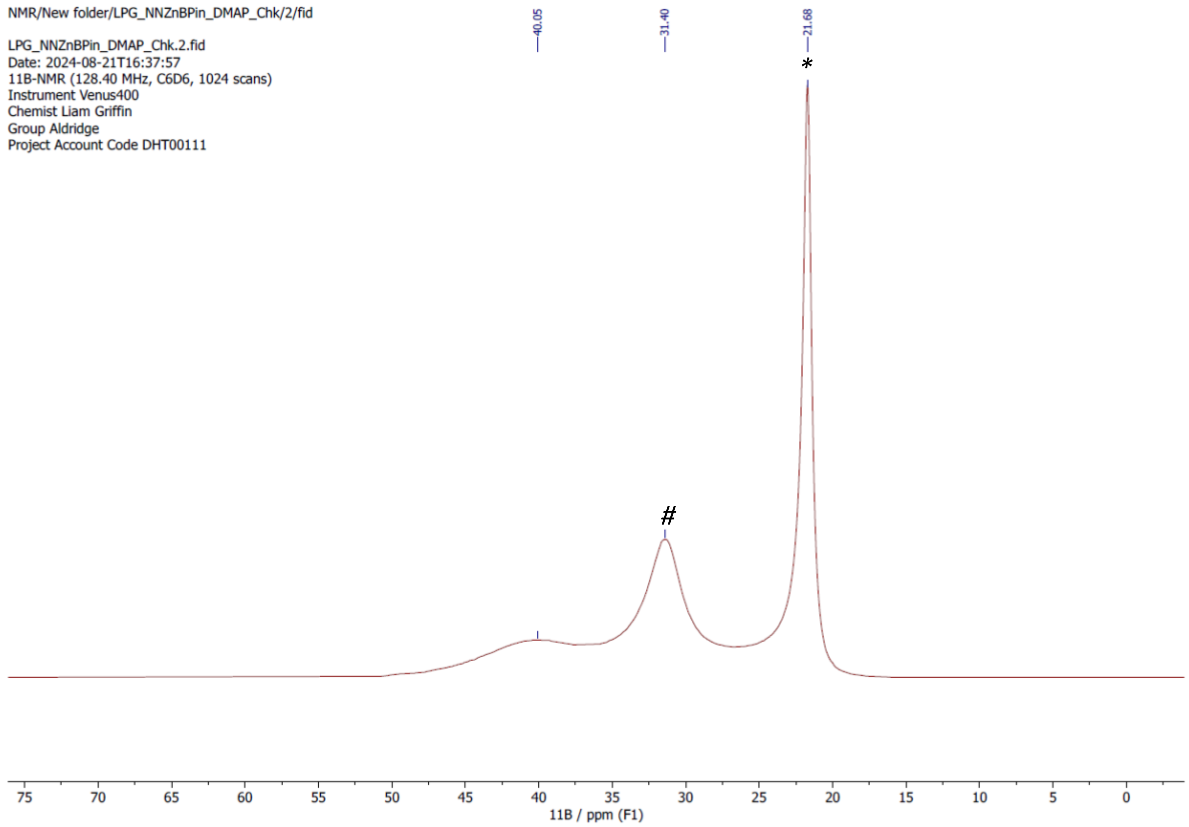

Fig S6. *In situ ^{11}B NMR spectrum of (Nacnac^{Mes})Zn(DMAP)Bpin (**2**). ^{t}BuOBPin by-product marked * and residual B_2pin_2 marked #.*

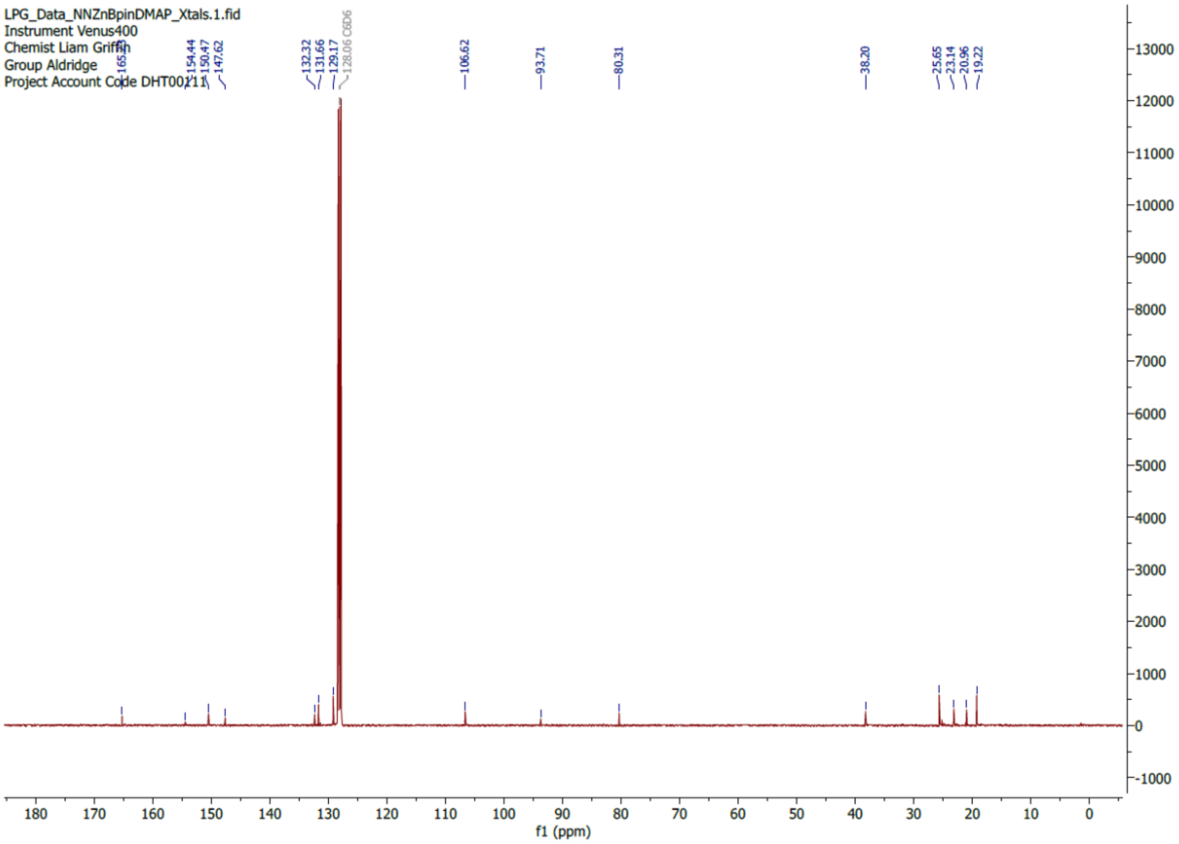

Fig S7. *^{13}C NMR spectrum of (Nacnac^{Mes})Zn(DMAP)Bpin (**2**).*

(Nacnac^{Mes})Zn(DMAP)I (3)

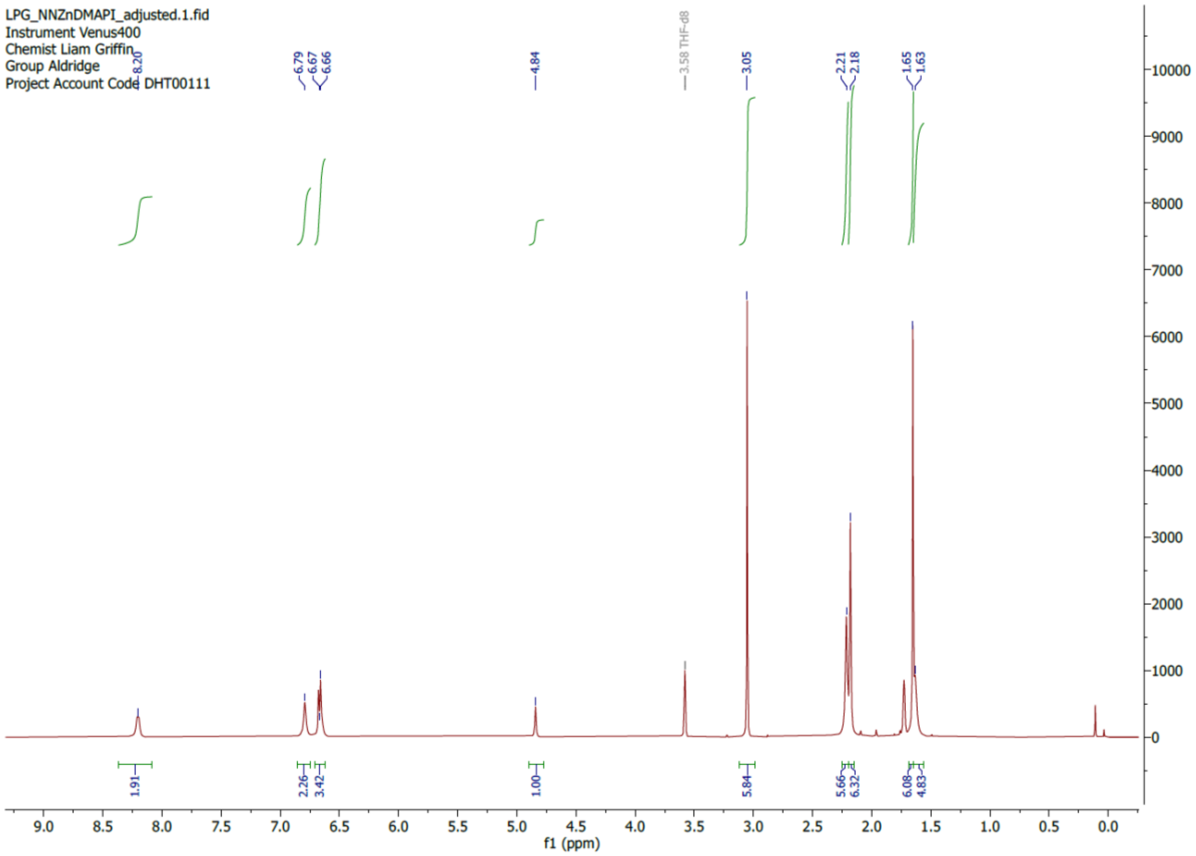

Fig S8. *^{1}H NMR spectrum of (Nacnac^{Mes})Zn(DMAP)I (**3**).*

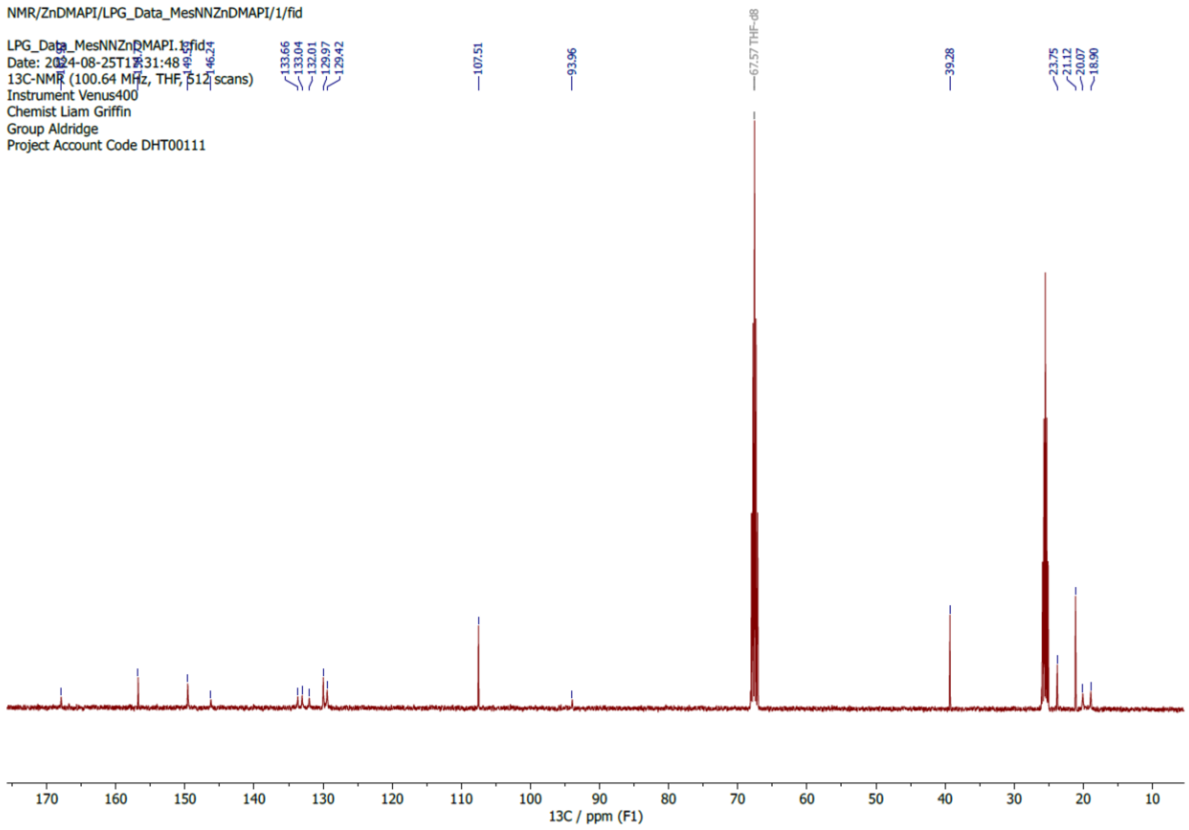

Fig S9. *^{13}C NMR spectrum of (Nacnac^{Mes})Zn(DMAP)I (**3**).*

[(Nacnac^{Mes})ZnOBpin]$_2$ (4)

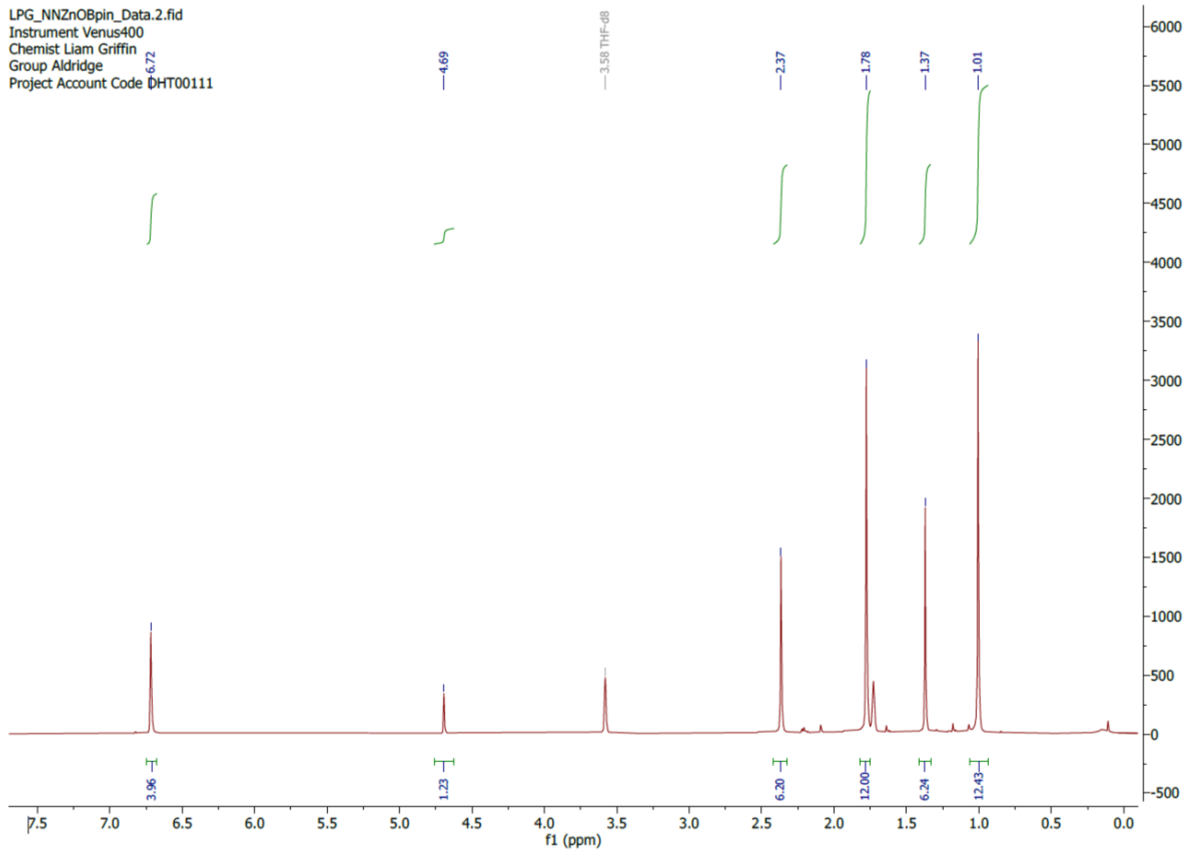

Fig S10. *^{1}H NMR spectrum of [(Nacnac^{Mes})ZnOBpin]$_2$ (**4**).*

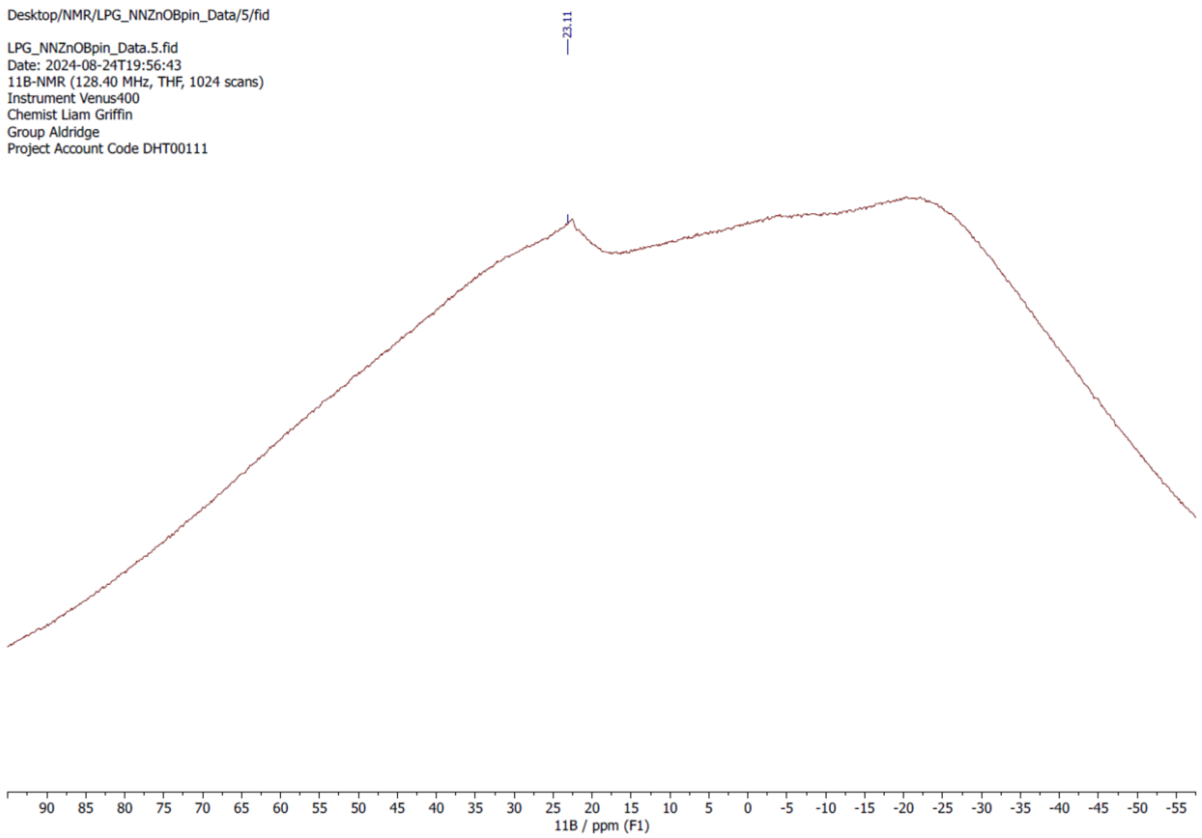

Fig S11. *^{11}B NMR spectrum of [(Nacnac^{Mes})ZnOBpin]$_2$ (**4**).*

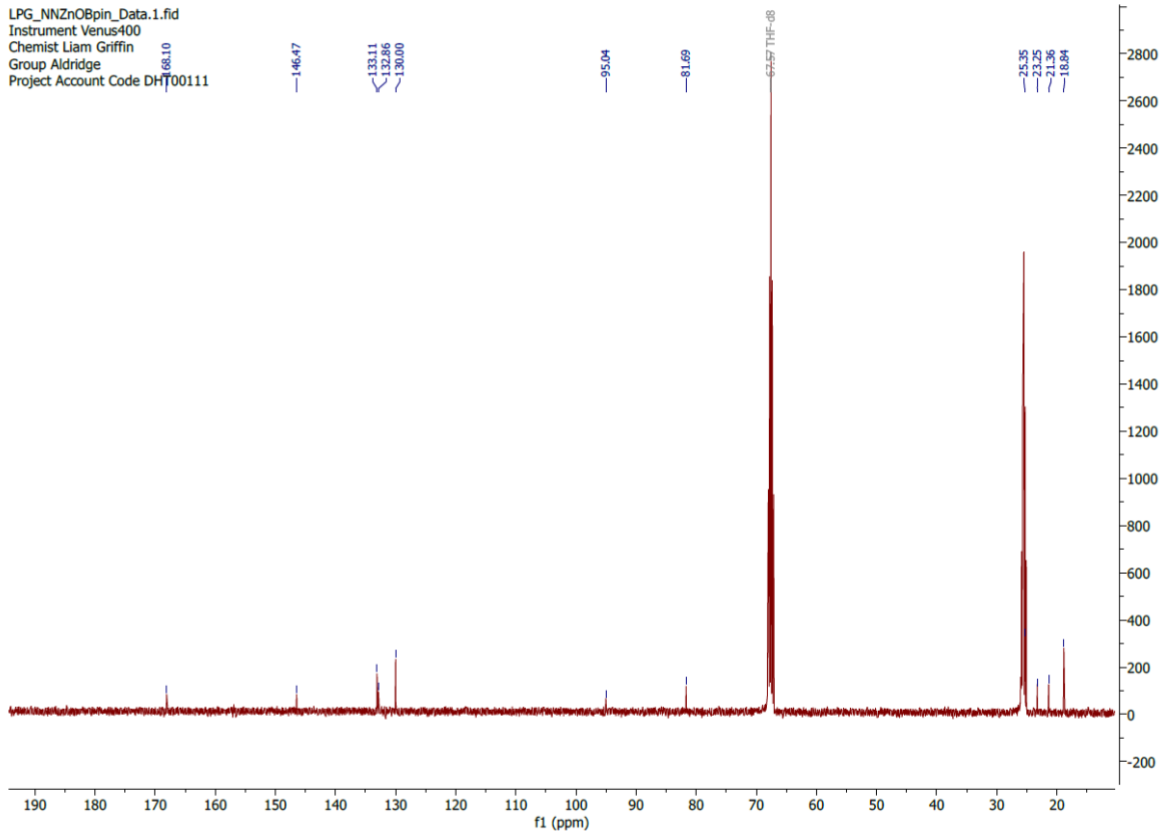

Fig S12. *^{13}C NMR spectrum of [(Nacnac^{Mes})ZnOBpin]$_2$ (**4**).*

(Nacnac^{Mes})ZnOB{(NDippCH)$_2$} (5)

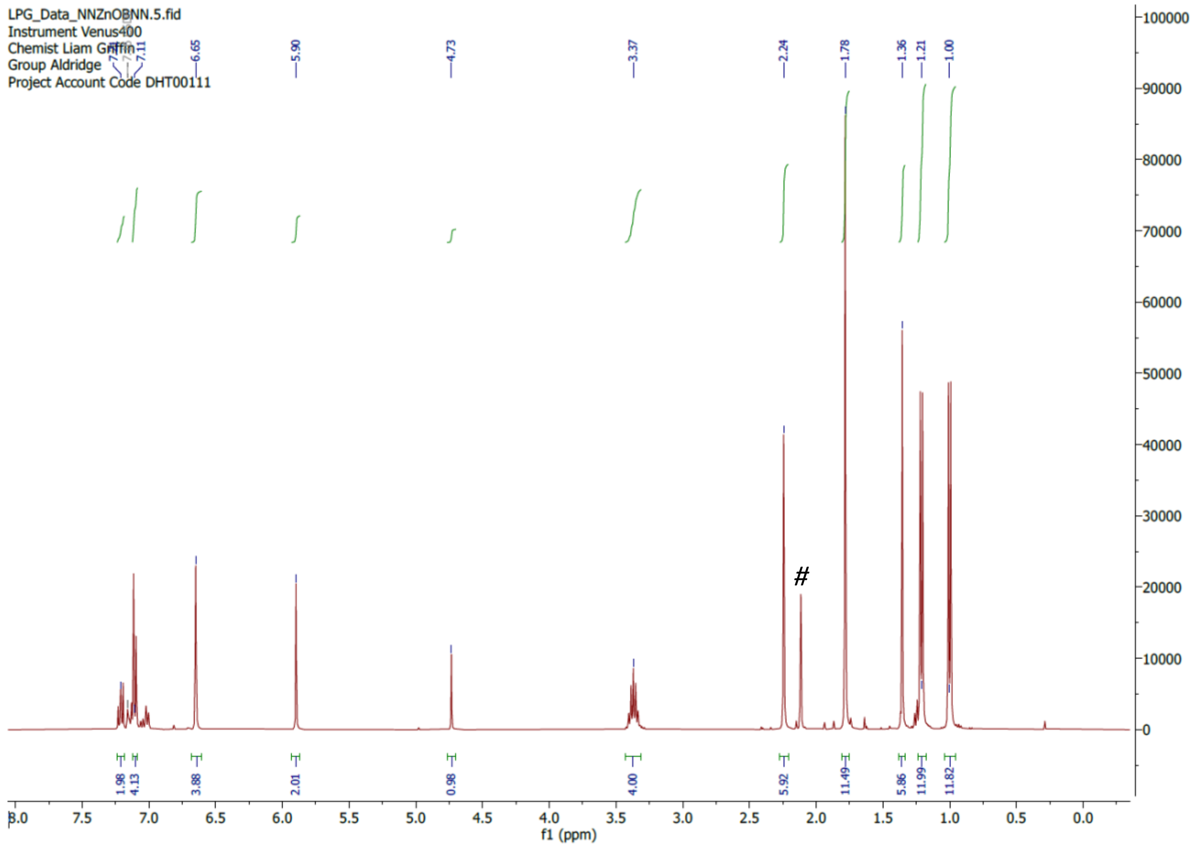

Fig S13. *^{1}H NMR spectrum of (Nacnac^{Mes})ZnOB{(NDippCH)$_2$}. Residual toluene marked #.*

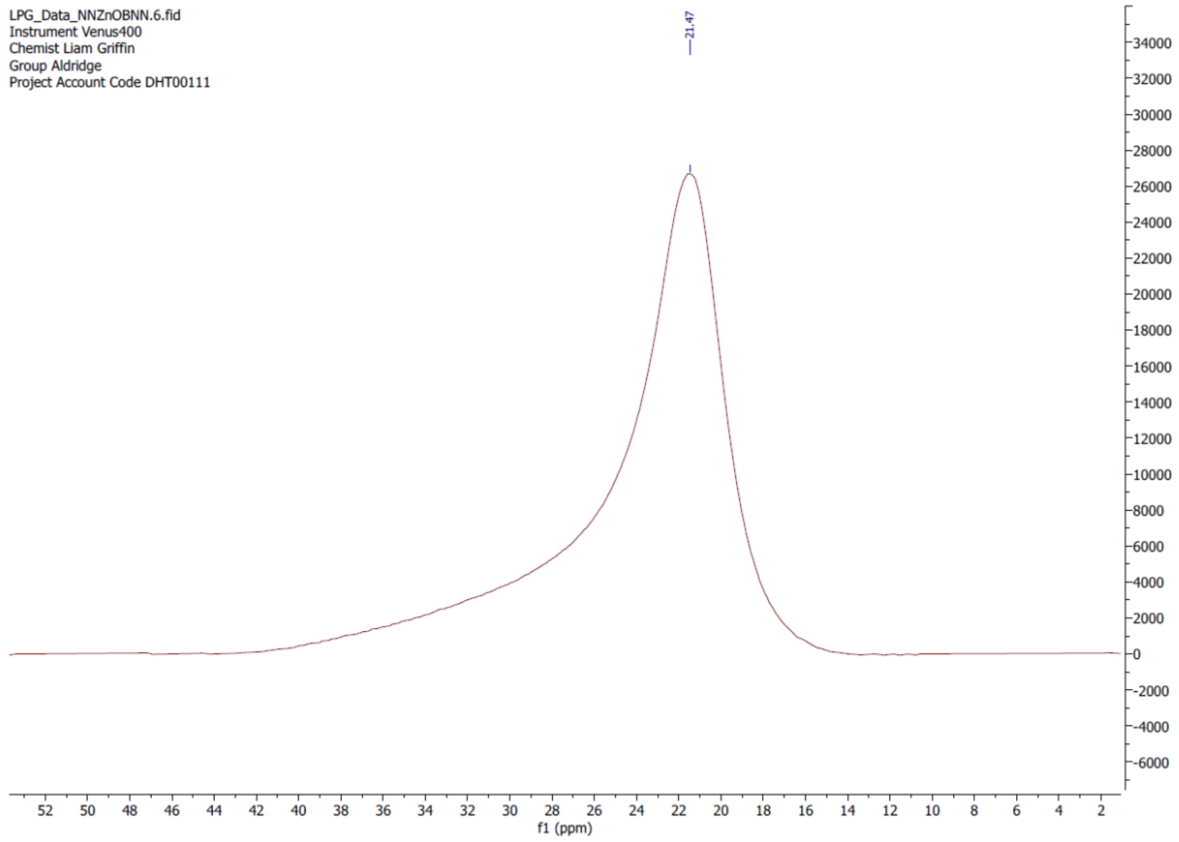

Fig S14. *^{11}B NMR spectrum of (Nacnac^{Mes})ZnOB{(NDippCH)$_2$}.*

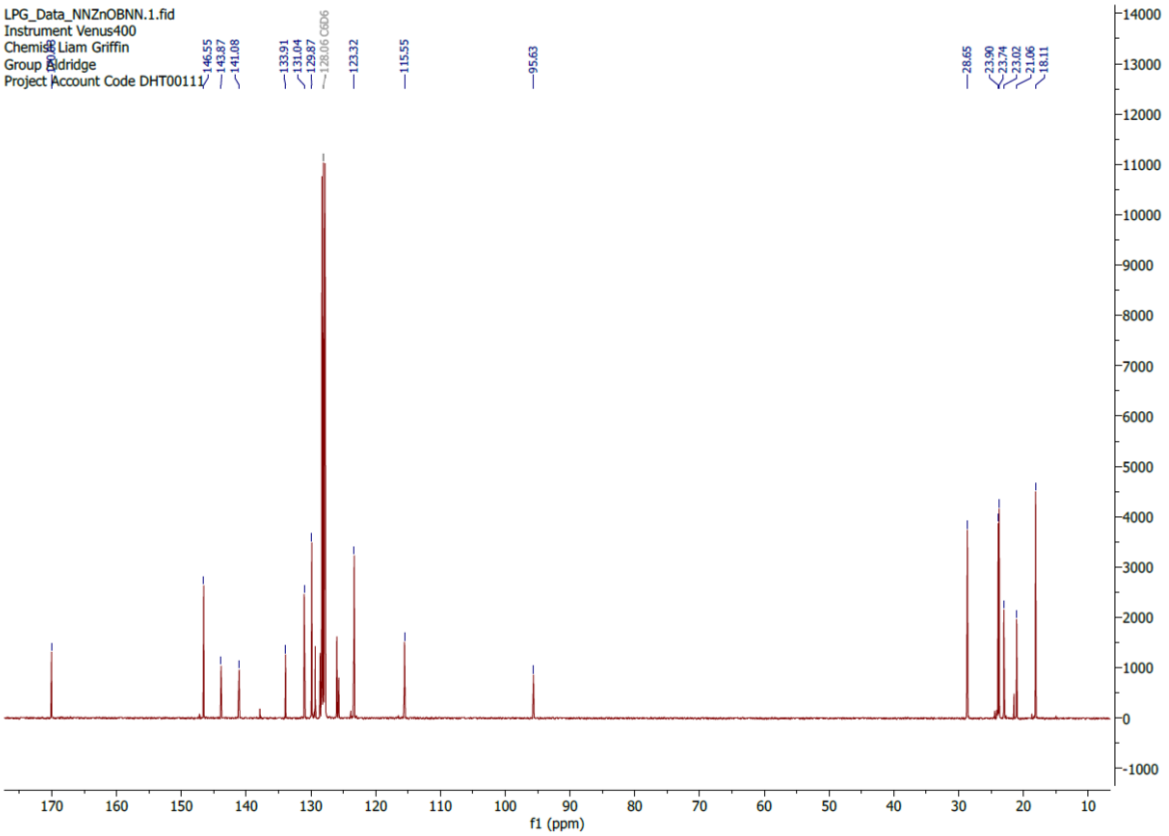

Fig S15. *^{13}C NMR spectrum of (Nacnac^{Mes})ZnOB{(NDippCH)$_2$}.*

{(HCDippN)$_2$}BOBpin (6)

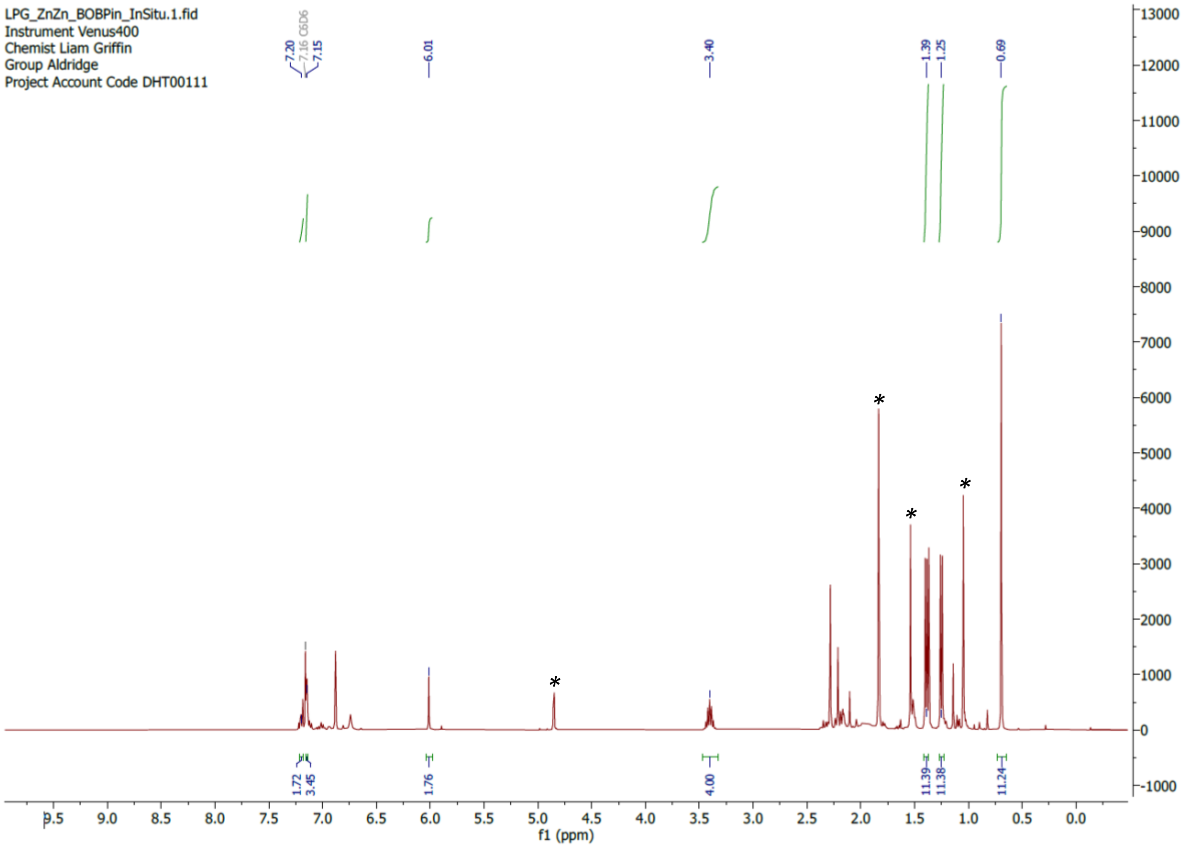

Fig S16. *In situ ^{1}H NMR spectrum of {(HCDippN)$_2$}BOBpin (**6**). Diagnostic signals of [(Nacnac^{Mes})Zn]$_2$ are marked *.*

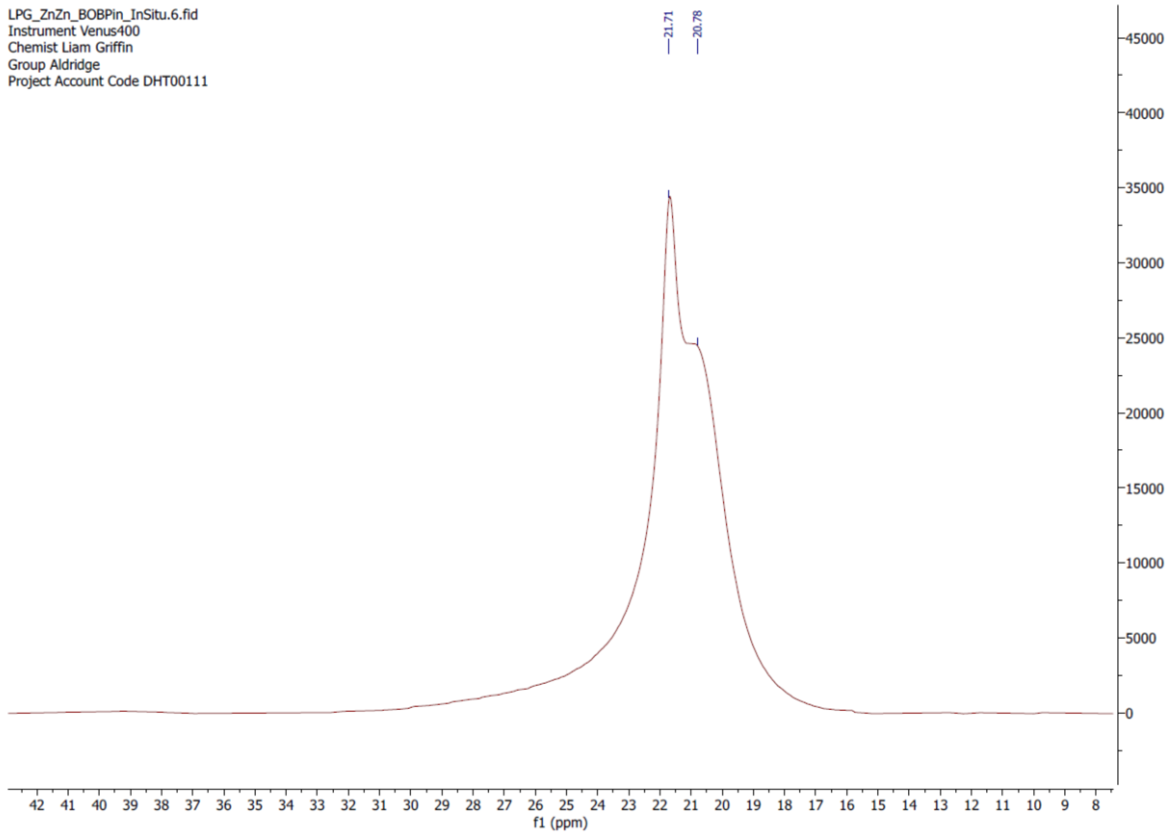

Fig S17. *In situ ^{11}B NMR spectrum of {(HCDippN)$_2$}BOBpin (**6**).*

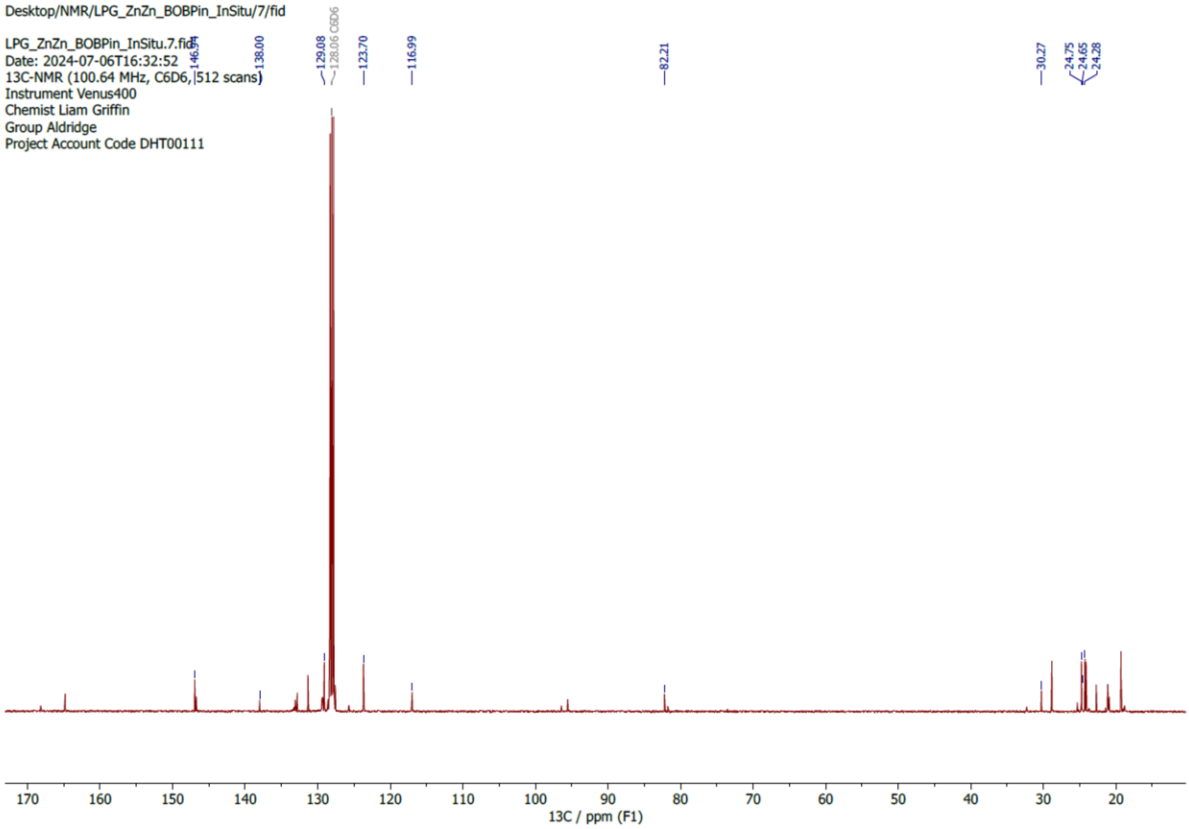

Fig S18. *In situ ^{13}C NMR spectrum of {(HCDippN)$_2$}BOBpin (**6**), in the presence of [(Nacnac^{Mes})Zn]$_2$.*

5. Computational details

Gas phase geometry optimizations and frequency analyses were carried out using the ORCA (5.0.4) software package,[S11,S12] using the R2-Scan-3C method.[S13] The optimized structures were confirmed to be minima on the potential energy surface by the absence of imaginary frequencies. Single point calculations were performed using the ωB97X-D4 functional and Def2-TZVP basis set.[S14-S16] Natural bonding orbital (NBO) analyses were carried out using the NBO 7.0 program.[S17,S18] Atoms in molecules (AIM) analyses were conducted using Multiwfn software package.[S19] AIM bonding classifications have been made in accordance with the literature precedent.[S20] All iso-surfaces have been rendered at 0.05, unless otherwise stated.

(Nacnac^{Mes})ZnBpin

LUMO

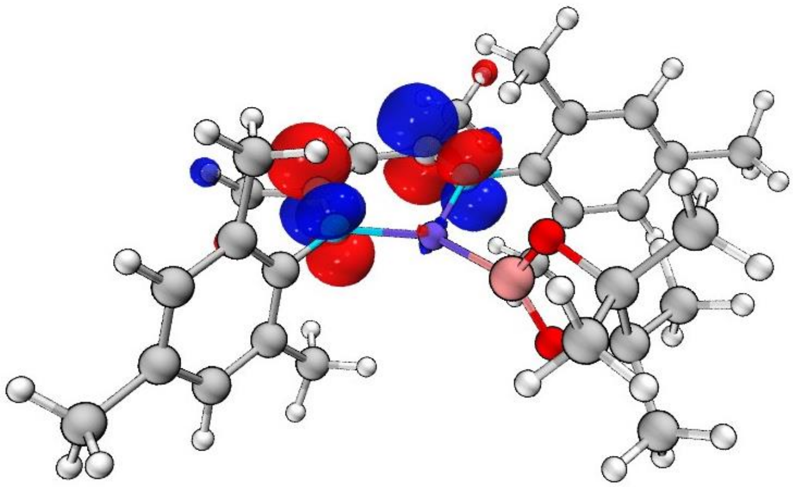

Fig S19. *Ligand based LUMO of (Nacnac^{Mes})ZnBpin (**1**), E = 1.2292 eV.*

HOMO

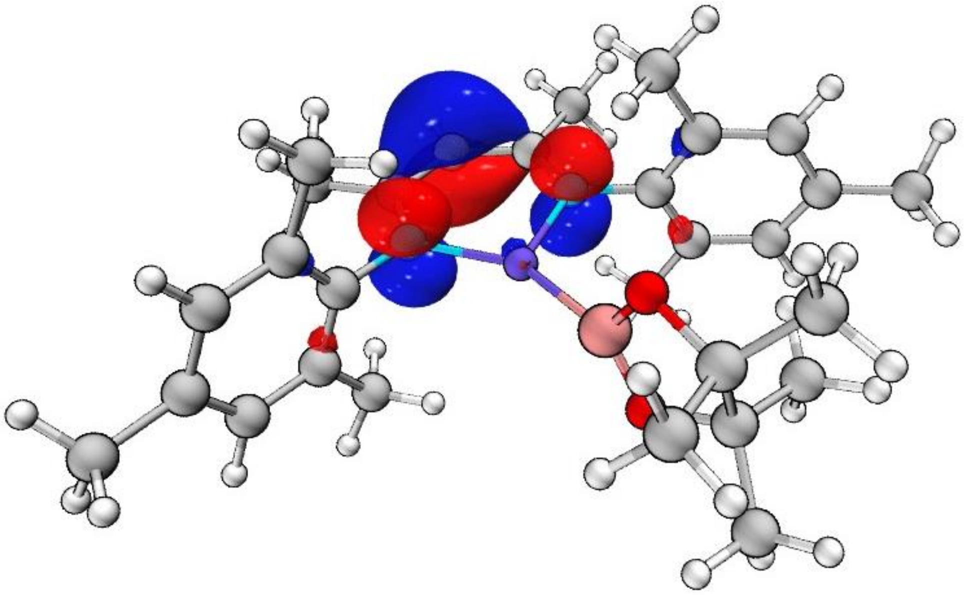

Fig S20. *Ligand based HOMO of (Nacnac^{Mes})ZnBpin (**1**), E = -7.8818 eV.*

Zn-B bonding contributions

HOMO-2

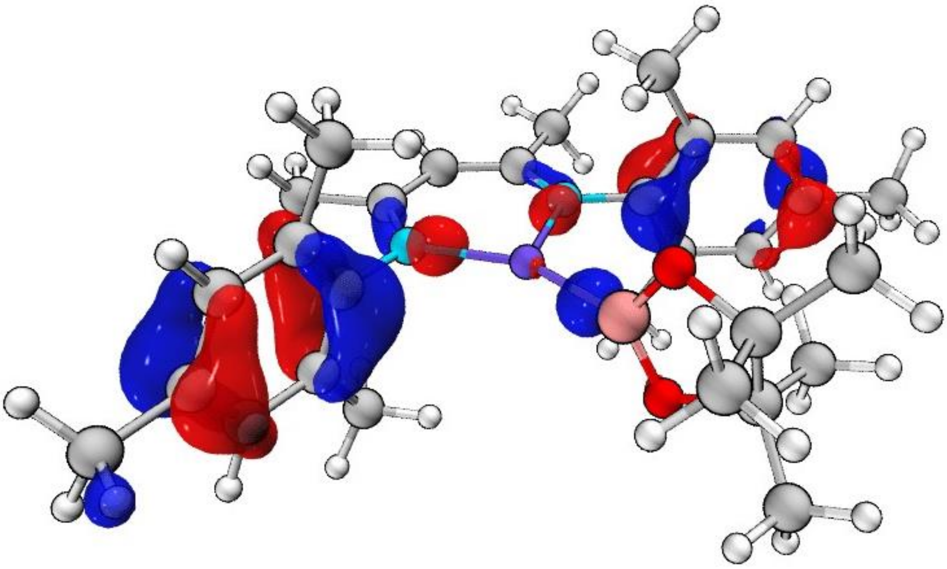

Fig S21. *HOMO-2 of (Nacnac^{Mes})ZnBpin (**1**), featuring a small region of electron density between B and Zn, E = -8.6125 eV.*

HOMO-3

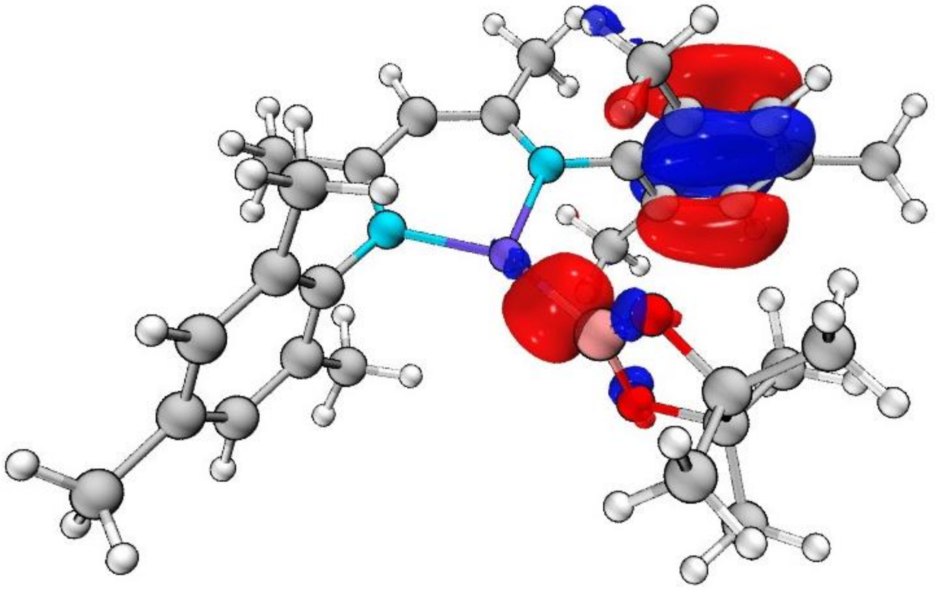

Fig S22. *HOMO-3 of (Nacnac^{Mes})ZnBpin (**1**), featuring a significant region of electron density between B and Zn, E = -8.8903 eV.*

HOMO-4

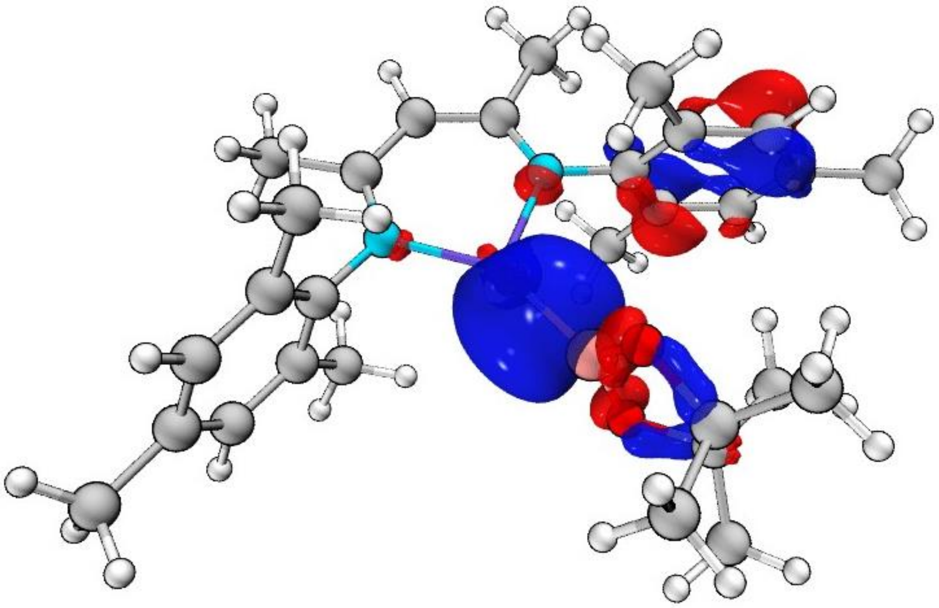

Fig S23. *HOMO-4 of (Nacnac^{Mes})ZnBpin (**1**), featuring the most significant region of electron density between B and Zn, E = -8.9182 eV.*

NBO analysis

One conventional NBO was located for the Zn-B interaction.

1.96172 electrons

21.75% (0.4664 electrons) Zn: s 96.5%, p 2.4%, d 1.1%

78.25% (0.8846 electrons) B: s 47.6%, p 52.4%

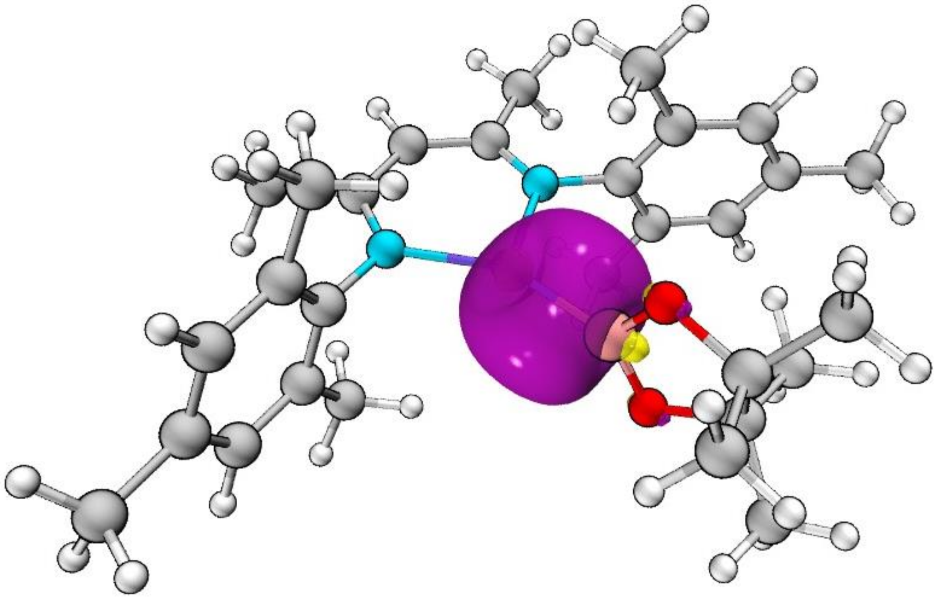

Fig S24. *NBO corresponding to the Zn-B bonding interaction in (Nacnac^{Mes})ZnBpin (**1**).*

WBI: 0.592

NPA charges

Zn: 1.47012 e

B: 0.46825 e

Bader charges

Zn: 0.879 e

B: 1.352 e

QTAIM

**ρ**	0.1001319145E+00
**$\nabla^2\rho$**	0.5864727743E-01
**G(r)**	0.5672856343E-01
**K(r)**	0.4206674407E-01
**V(r)**	-0.9879530750E-01
**E(r)**	-0.4206674407E-01
**ELF**	0.5441836390E+00
**ε**	0.084374

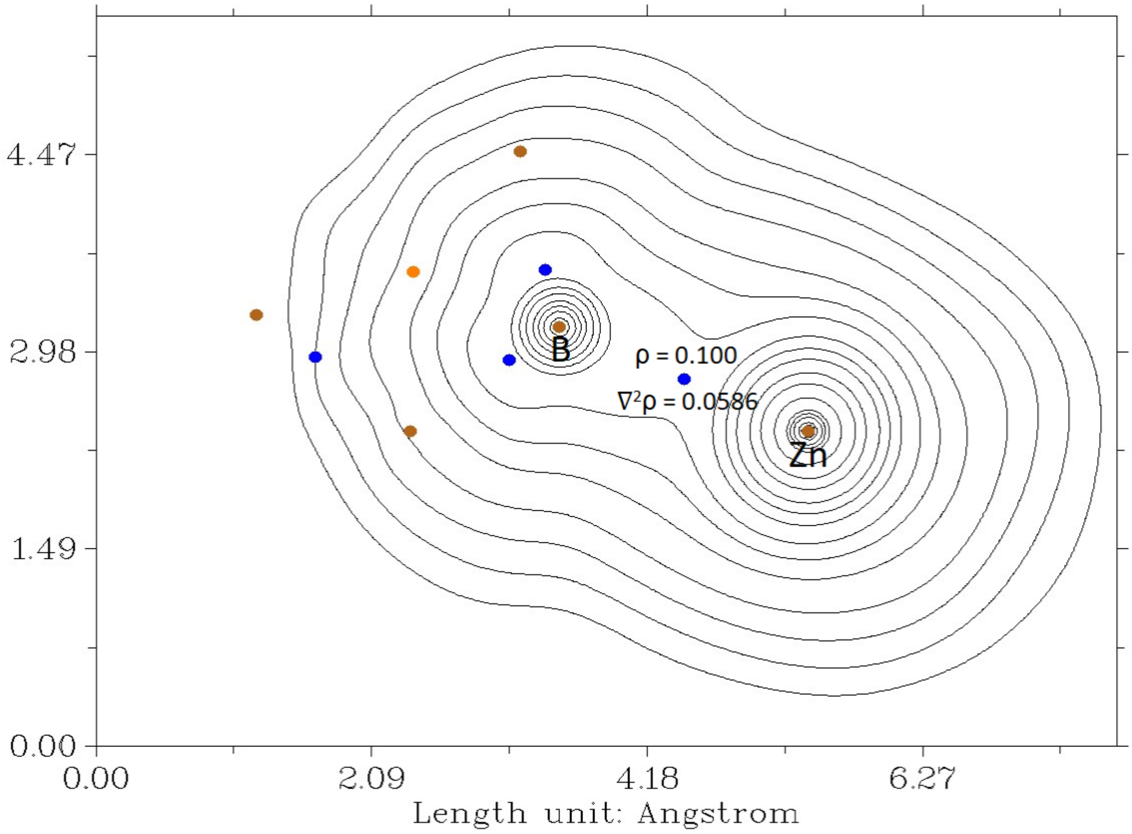

Fig S25. *Contour plot of electron density in the region around the Zn-B bond in (Nacnac^{Mes})ZnBpin (**1**).*

(Nacnac^{Mes})Zn(DMAP)Bpin (2)

LUMO

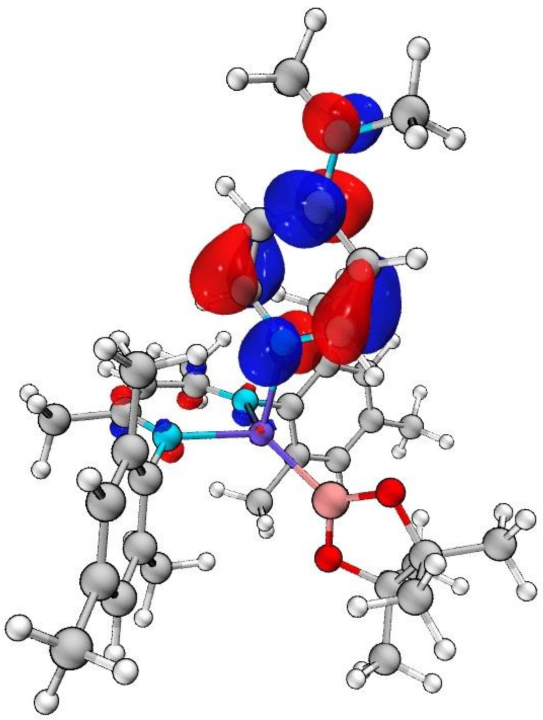

Fig S26. *DMAP-based LUMO of (Nacnac^{Mes})Zn(DMAP)Bpin (**2**), E = 1.3777 eV*

HOMO

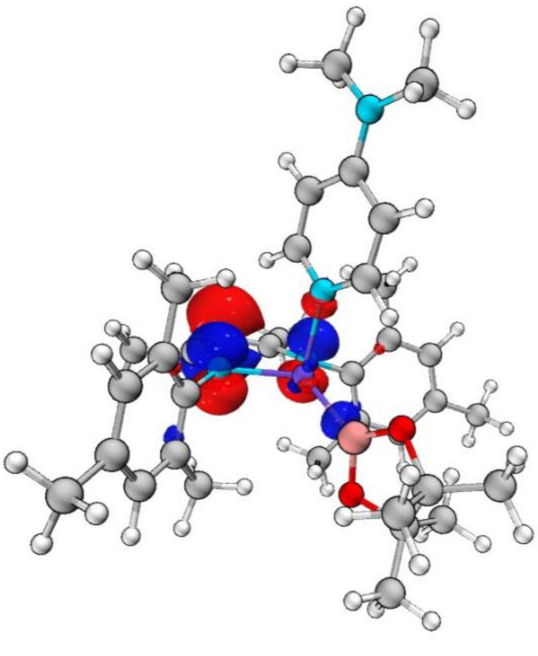

Fig S27. *Primarily ligand-based HOMO of (Nacnac^{Mes})Zn(DMAP)Bpin (**2**), with a small amount of electron density between B and Zn, E = -7.1873 eV.*

Zn-B bonding contributions

HOMO-2

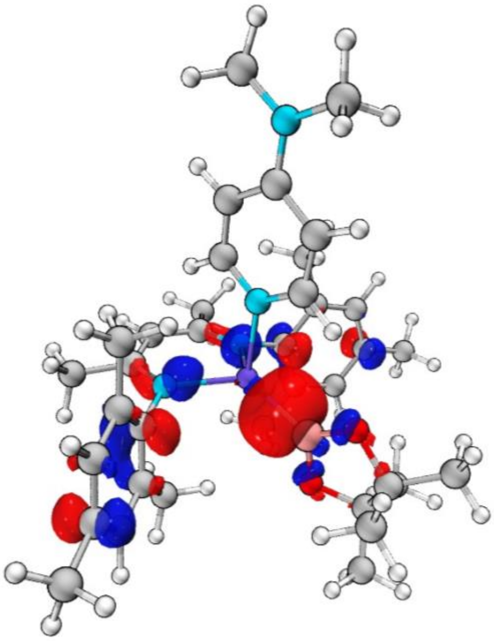

Fig S28. *HOMO-2 of (Nacnac^{Mes})Zn(DMAP)Bpin (**2**), featuring a significant contribution to Zn-B σ-bonding, E = -8.1452 eV.*

HOMO-3

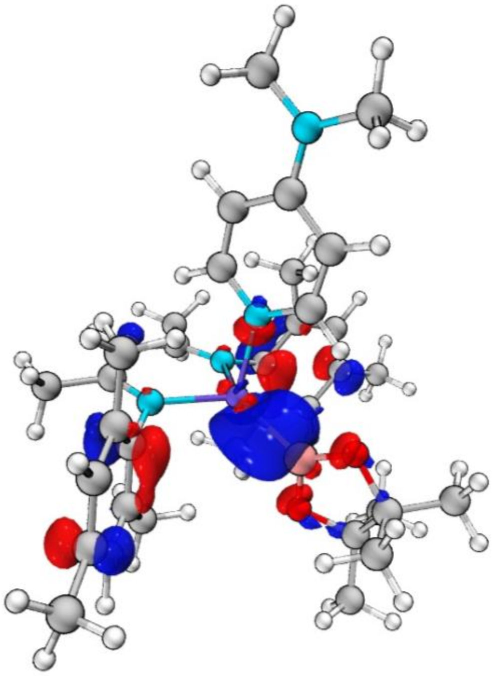

Fig S29. *HOMO-3 of (Nacnac^{Mes})Zn(DMAP)Bpin (**2**), featuring a significant contribution to Zn-B σ-bonding, E = -8.2358 eV.*

NBO analysis

No NBO was located for the Zn-B bonding interaction. This is instead described as a second-order perturbation interaction between a B-based lone pair and a Zn based vacant orbital, with an overall stabilization energy of 232.81 kcal/mol.

B-based lone pair – 1.64650 electrons: s 49.3%, p 50.67%

Zn vacant orbital – 0.44130 electrons: s 98.7%, p 1.0%, d 0.3%

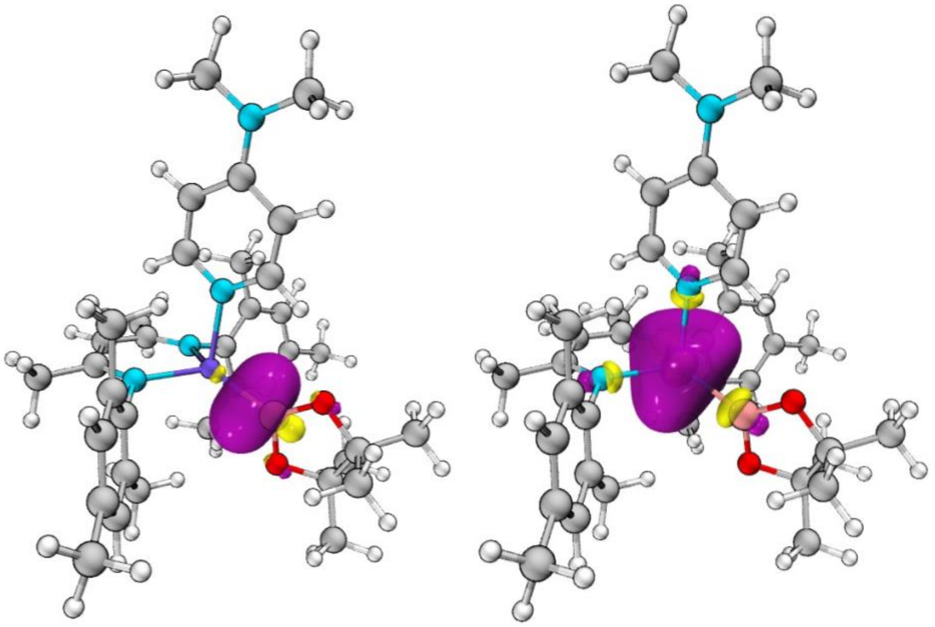

Fig S30. *NBO orbitals for (Nacnac^{Mes})Zn(DMAP)Bpin (**2**) calculated to interact by Second Order Perturbation Theory, leading to a Zn-B σ-bonding interaction. Left, B-based lone pair, Right, Zn-based vacant orbital.*

WBI: 0.496

NPA charges

Zn: 1.56613 e

B: 0.41854 e

Bader charges

Zn: 0.975 e

B: 1.272 e

<u>QTAIM</u>

**ρ**	0.9544211041E-01
**∇²ρ**	0.9014936624E-01
**G(r)**	0.6015711525E-01
**K(r)**	0.3761977370E-01
**V(r)**	-0.9777688895E-01
**E(r)**	-0.3761977370E-01
**ELF**	0.4750102996E+00
**ε**	0.058560

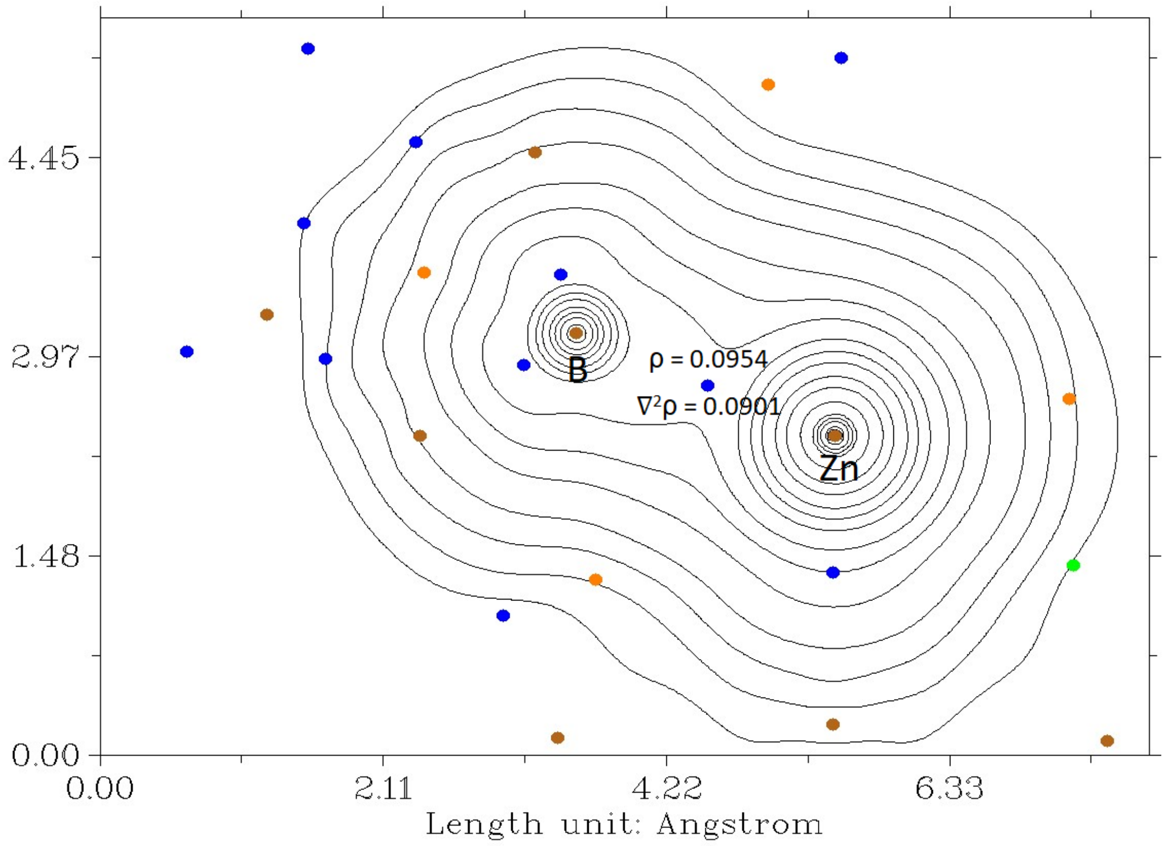

Fig S31. *Contour plot of electron density in the region around the Zn-B bond in (Nacnac^{Mes})Zn(DMAP)Bpin (**2**).*

Table S2. *Key QTAIM parameters (in a.u.) relating to the BCPs of the Zn-B bonding interactions for zinc-boryl species **1** and **2**.*

	(Nacnac^{Mes})ZnBpin (**1**)	(Nacnac^{Mes})Zn(DMAP)Bpin (**2**)
**ρ**	0.1001	0.09544
**∇²ρ**	0.05865	0.09015

7. Optimised XYZ coordinates

**Nacnac^{Mes}ZnBpin (1): -3195.348038663298 E_h**			
Zn	-1.00569597992652	8.36829931809950	15.63429840251350
O	0.23312213009687	8.82138844931929	12.91695432176124
O	0.52007541007981	6.66428836442517	13.61985128564829
N	-1.00132060882704	8.98680513854936	17.48706083474262
N	-2.98397560074018	8.17560082294936	15.52626010096209
C	-3.82584362761764	8.48661489253424	16.50201497363231
C	0.27947824933209	9.24194269732946	18.05966785395151
C	-3.46404433021647	7.68090421113294	14.28142371969911
C	-2.09955684484563	9.18168116580471	18.21976143433770
C	0.86515508562621	10.50775852417875	17.91062008689743
C	-3.40577565560078	8.95756409529206	17.75964034207281
H	-4.19559568496607	9.17288776875349	18.46873064547313
C	0.96903761986552	8.20356947631389	18.70082616086399
C	-5.31030102528431	8.34069367031991	16.26740517725800
H	-5.62208463098462	8.94142523664205	15.40560004437338
H	-5.88284359683960	8.65155586413264	17.14269733518252
H	-5.56550887310249	7.30293616430865	16.02633832431562
C	0.98883870833172	8.15462424257073	11.85552865940697
C	-3.64647705186926	8.56705293370207	13.20887074662354
C	2.12870611863673	10.72821612053757	18.45538848032458
H	2.58266030170770	11.71221954637801	18.35024483314607
C	2.82588523452401	9.72211164650550	19.12392416834940
C	-3.35937440624044	10.03143294109755	13.38047222482478
H	-2.28290962635983	10.18931165992444	13.53062775621891
H	-3.86960279493573	10.45249679558921	14.25332004757756
H	-3.66177085846881	10.59461133397619	12.49374523406720
C	2.23170623746087	8.46600218474290	19.23138906501148
H	2.76606100526013	7.66423628153838	19.73788484041198
C	-3.63946841055333	6.29994085257612	14.10533858610848
C	4.20210410669543	9.97759265939289	19.67750303863014
H	4.96499938477502	9.86933477596887	18.89661662063115
H	4.44673722145007	9.27179505412282	20.47645313837126
H	4.28795363921820	10.99223159360665	20.07871758419871
C	-1.94189946723816	9.67975989421774	19.63715538683082
H	-1.33342618185558	8.98590239961452	20.22745636879637
H	-2.91203616060652	9.79933243346003	20.12135277569335
H	-1.41468466011727	10.63992671532522	19.65507029765383
C	0.73778903605291	6.64510792256562	12.17264868152541
C	0.14709628785161	11.59027223124144	17.15500716485876
H	0.71435088699354	12.52422152384613	17.17935720872944
H	-0.85367516445567	11.78192751575098	17.55761340447506
H	0.00837957513403	11.30265187491263	16.10435787225475
C	0.36244534525873	6.83121960931995	18.78743644526742
H	1.00078531357699	6.15846248631201	19.36579675140064
H	0.23327981850129	6.40125201527618	17.78547917198924

H	-0.63186491435181	6.84660215836907	19.24738622244431
C	-4.06062734999684	8.05480853953977	11.97976339516872
H	-4.21032914424376	8.74183593281979	11.14847199228339
C	-3.32804713498226	5.34782570386731	15.22523578652070
H	-3.59362019477010	4.32362007682671	14.95098716579851
H	-3.85143537262757	5.60608696496562	16.15209415423432
H	-2.25462285626204	5.37161412988234	15.45576953485031
B	0.07304448756951	7.92702078893298	13.95840084221468
C	-4.05920697301049	5.83025511932885	12.86182836852453
H	-4.20118919368124	4.75946534422299	12.72531708326651
C	2.44717334875777	8.57193069660631	12.02548239821174
H	2.50886617339186	9.66350991338173	11.98765709434612
H	3.07651032636746	8.16148502024486	11.22885191362045
H	2.83878400900518	8.24260417030653	12.99255647722442
C	0.45080207650664	8.62408617740779	10.51437488118228
H	-0.63061134178426	8.48404400542419	10.44969500250764
H	0.92828452643038	8.07644501509085	9.69388056646429
H	0.66704568073475	9.68894959713337	10.38562408499210
C	-4.27870160251624	6.69111208084301	11.78765328700445
C	1.90288007569518	5.72087170226832	11.86398665901859
H	2.79574565993803	5.99527596060713	12.42946067267719
H	2.14065537036363	5.74717890579368	10.79440644423122
H	1.63379180553312	4.69358449202173	12.12732160136328
C	-4.68118532347664	6.15632897055789	10.43970265670045
H	-5.28049991822012	6.88387713391947	9.88410960345592
H	-5.26214419480631	5.23405474856243	10.53358303092309
H	-3.79661350826854	5.92678277334761	9.83177579487694
C	-0.55189104368806	6.10996662259340	11.55342687561135
H	-0.77000315874329	5.13040721296623	11.98944954521262
H	-0.45902216422676	5.99436058083086	10.46809608281842
H	-1.39884362541463	6.76814635717840	11.77174318518715

Nacnac^{Mes}ZnOB{(NDippCH)$_2$} (5): -4007.029880121355 E_h

Zn	1.77209368059339	8.07311725809021	15.09667972475072
O	1.36650550644669	8.30270856516209	13.34274955456828
N	3.61564948723860	7.80273374126823	15.68911459300305
N	0.82016550544546	7.62751184508411	16.71614580906611
N	-0.01990301896639	10.00950916287920	12.06662725842504
N	1.28556264005621	8.56357797229134	10.86541686442202
C	4.69001897398264	7.94824184130176	14.75962943057442
C	5.51515992922284	9.08146830864706	14.80962973594603
C	1.49197808428461	7.26148499284366	17.81424249839451
C	3.86720329345713	7.31729339118173	16.90203945706116
C	-0.75423942721806	10.73291543767099	13.04317901783591
C	4.87702005432260	6.96195722069282	13.78052830224434
C	-0.60775639293589	7.59650064149891	16.75924920628608
C	-1.31662306328746	8.76434727189238	17.06268246041562
C	2.88504888430707	7.12585638269352	17.89098868138104
H	3.25806796598992	6.77854170036045	18.84623406512002
C	5.27650494980375	10.16843909914266	15.82106079840106

H	4.21590483169879	10.43242473453470	15.87629222814001
H	5.84341412043759	11.06637740538387	15.56152537920866
H	5.57546894438557	9.86926717318692	16.83337432328468
C	5.27588502102586	6.89636462914789	17.24649101398751
H	5.61864914838416	6.13456862342055	16.53693268794416
H	5.32587052360669	6.48687568061776	18.25613988782268
H	5.98161612315752	7.72803808877052	17.16611072164149
C	2.29151579363558	7.65811094634765	10.43600795097646
C	-0.17718080761309	10.26287161711502	10.69307882349267
H	-0.85340264636654	11.02568815488026	10.33288573811368
C	6.56415583701451	9.17908738598511	13.89575646710691
H	7.20950758896698	10.05546846987835	13.92959258210126
C	3.49843947740264	8.16478680932075	9.91307957864631
C	0.70658060124963	6.94157968977419	19.06426566996504
H	0.03210283138662	7.76541473393862	19.32000090069417
H	1.37416118575120	6.75124450935122	19.90539317958655
H	0.07084678611829	6.06224280902954	18.91346272296993
C	-1.27642164579708	6.39303405089054	16.49051387007683
C	5.93346353455527	7.10348130989805	12.88349808327244
H	6.07281696913556	6.34488105711096	12.11637564007251
C	0.59951931996091	9.41227955653025	9.98611466011858
H	0.70158521255100	9.32527091685278	8.91296570042303
C	-2.70762730190231	8.69571976780864	17.14692785334080
H	-3.26300462928344	9.60051601636510	17.38593804280635
C	-2.06669445263916	10.32629303560555	13.34900076951729
C	2.06752092917377	6.27362908696819	10.56005518981009
C	-0.17101215306035	11.85672528392986	13.65501504406458
C	3.75842387316118	9.66213232464734	9.89215800083883
H	2.82292856466026	10.16357735571861	9.61935519843585
C	3.06827190875428	5.40226055061084	10.12684888215428
H	2.92135878775355	4.32972010101117	10.20629880076456
C	3.95411314278257	5.77963910863267	13.70312713367153
H	3.86781746173416	5.26243521050568	14.66598400376226
H	4.30083703607981	5.06838518580404	12.94972043267317
H	2.94659216317115	6.10110648663425	13.40680225446494
C	-3.39775732249191	7.50466703483411	16.93189330053000
C	4.46581936594266	7.25439971531786	9.48571263460324
H	5.40336381642570	7.61628398037640	9.07500364708017
C	-2.66474247219893	6.36458263759720	16.59929187578624
H	-3.18960297373295	5.43231726142043	16.39813171062026
C	6.79334709761805	8.19880908689507	12.93121993061535
C	4.25350144256477	5.88572152457404	9.58810438421271
H	5.01719581578589	5.19127732198192	9.24764094901641
C	0.74910115129283	5.75684031231684	11.11089294770564
H	0.48454245068384	6.38841581522578	11.96775275694785
C	-2.67279690569914	9.12349660139833	12.64904880979682
H	-2.24778794615910	9.08411116531873	11.63871799340595
B	0.91767548346090	8.90713307594080	12.23878374175500
C	-2.27569658519296	7.82909450821320	13.37354098382239
H	-1.18851836529513	7.73163616350687	13.45671390238629
H	-2.66097918897686	6.95344435541312	12.83692990103138

H	-2.68935387090205	7.82294633459427	14.38613136146491
C	0.81345903004320	4.31477658285622	11.61907864246420
H	0.94193373417456	3.59738518597792	10.80008983762849
H	-0.12539145553646	4.06245472246749	12.12389954939622
H	1.63226876553790	4.16911811039522	12.33239467923955
C	-0.58692308315431	10.05518374859213	17.29462680677016
H	0.19322427050255	9.95350199107229	18.05759855936460
H	-1.27839180964261	10.84271031699713	17.60197073063119
H	-0.09520235838538	10.39469841256519	16.37376404519366
C	-4.90038162800373	7.45964875753917	16.98914223428702
H	-5.30138500334102	8.27571005885600	17.59692259093909
H	-5.25621357547604	6.51284258986791	17.40699182871699
H	-5.33014369931980	7.55409403097139	15.98352317833097
C	-2.77045002539485	11.04270802530881	14.31759933342557
H	-3.78146312050653	10.74591414355507	14.57918930185187
C	-0.50721881919855	5.17159476736260	16.07071678336314
H	-1.17717657837363	4.31968994243826	15.93033800741539
H	0.26046712462375	4.89036267422421	16.80041600254391
H	0.01635382116123	5.35017022124383	15.12165654763733
C	1.23375188661575	12.29761969730995	13.28897258866543
H	1.46325358625114	11.86786687955432	12.30703151071396
C	4.14019986782764	10.15118531170080	11.29876689735639
H	3.39092622670136	9.86232472171439	12.04158103026599
H	4.23801157751387	11.24360327127860	11.31139634097495
H	5.09401261262317	9.71411437969078	11.60937467291683
C	-0.91329589666709	12.54754609325839	14.61555536372044
H	-0.48559922319707	13.41860221152663	15.10431401537396
C	4.81740391836010	10.09342524936681	8.87624112716375
H	5.81874495050685	9.74736123534991	9.15815061144486
H	4.85736207356555	11.18668121907239	8.82726936356248
H	4.59585527598027	9.71467359728650	7.87263023070392
C	-2.19897394693483	12.14226037720955	14.94661781593183
H	-2.76476704680747	12.69273669167171	15.69392942128826
C	7.89378810776611	8.35558848801834	11.91785251765525
H	7.54096385304162	8.92556162964884	11.04800597858413
H	8.23964317485524	7.38434864277351	11.55241599637056
H	8.75058249590890	8.89330061897517	12.33504167801856
C	2.25295619163431	11.73479912980944	14.28965883718382
H	2.04584568772915	12.10378941399017	15.30217689292425
H	3.27333297093790	12.02771369213324	14.01680825984477
H	2.21369021533195	10.63905588485738	14.30227467485301
C	-0.36772372824453	5.89679049477568	10.06387533497799
H	-0.48317251072023	6.93542252000634	9.74253856367856
H	-1.32471907324905	5.55993276736850	10.47940303588602
H	-0.14217363635615	5.28437190826925	9.18271899534775
C	-4.19484572827726	9.20595049318874	12.51119247638568
H	-4.69627506231178	9.10943407204559	13.48132721837126
H	-4.55401899391795	8.38679663151796	11.87935918570378
H	-4.50962636490736	10.15189216522061	12.05766954858662
C	1.37399690208347	13.81930908002849	13.17806101355828
H	0.60881178139301	14.24108577210339	12.51854413272241

H	2.35791681048499	14.07334044361921	12.76913631929695
H	1.29113332786567	14.31168533953158	14.15340298613594
**{(HCDippN)$_2$}BOBpin (6): -1634.929067902274 E_h**			
N	-0.27767672420927	9.42334436577732	12.03597161894040
N	1.05407548357170	8.22094710087637	10.62463590420619
C	-0.89793908864084	10.01437076174841	13.17183385162329
C	2.03918285628917	7.37591528596370	10.03297344001831
C	-0.54052258376547	9.80678823130487	10.71140455350665
H	-1.27453139924964	10.56413597293417	10.47660534266072
C	3.31935417084815	7.89890552438230	9.78303549152910
C	0.24836159190121	9.09325738938099	9.87503711887983
H	0.30910242207340	9.12819814238430	8.79662364614057
C	-2.24180091904982	9.71523298427296	13.45954408182512
C	1.70865565492961	6.04569690665699	9.72964400163563
C	-0.13569569223634	10.85772078099751	14.00087555150896
C	3.66418047355870	9.32927259015195	10.15588224582716
H	2.72667225063541	9.89559564002169	10.20510778535828
C	2.70318525471720	5.22944404247613	9.18750101465614
H	2.47865556537268	4.19308556996301	8.95210625022234
C	4.28030373543085	7.04813471986027	9.23573695330397
H	5.28052117388928	7.42217609066823	9.03804973871230
C	3.97677174431658	5.72446319261221	8.94391803474320
H	4.73856149730736	5.07509775572343	8.52138591882470
C	0.32808191748939	5.49088630107379	10.02512426561503
H	-0.34995047530986	6.34179075635485	10.15760900566340
C	-3.07947771916563	8.83238050400592	12.55353684805544
H	-2.42812127725678	8.44344998928776	11.76395387729665
B	0.74320249395657	8.41037755333230	12.01093729487811
C	-3.66051098456920	7.62496869616465	13.29875032102023
H	-2.85683660886606	7.03086300828283	13.74049943517413
H	-4.22508925547413	6.99170972149974	12.60502082871517
H	-4.34489874138905	7.93362884971411	14.09724045171924
C	0.34572659276120	4.69837698639444	11.34176903529595
H	1.00814087072109	3.82888246153467	11.25660875141802
H	-0.66009794213644	4.34185045795120	11.59205745787653
H	0.70716915397828	5.31848014261426	12.16769194264242
C	-2.80596925563966	10.26444262107796	14.61044886116454
H	-3.84234896392283	10.04596248508724	14.85489062493070
C	1.28548054364669	11.26840627998264	13.65627092325660
H	1.57555032366145	10.75063383711345	12.73555213666610
C	4.30816222811118	9.37027074655575	11.55144261361346
H	3.66035806509453	8.91157072807682	12.30497419442841
H	4.51063820196659	10.40580065046590	11.84998547887850
H	5.25871547659314	8.82380241389997	11.54859551822005
C	-0.74028206818962	11.37009033247475	15.15117381226526
H	-0.17038437296873	12.02101332160834	15.80930365492193
C	4.56217636176267	10.01888992679359	9.12388721894623
H	5.57111454555915	9.59257647372857	9.10976449922550
H	4.66387350005949	11.08115450828855	9.37042150481447

H	4.14538458094499	9.93926867093296	8.11452048274983
C	-2.06089495068698	11.07431249791927	15.45826414447513
H	-2.51521694973932	11.48474732800939	16.35597073921612
C	2.28836997897480	10.87413116267311	14.74737115735774
H	2.05867566944093	11.36910888330626	15.69820091767124
H	3.30118364354111	11.17073199258351	14.45315805041331
H	2.29016662891271	9.79361527137177	14.91878563491658
C	-0.22382237184823	4.63266244240774	8.88145050393301
H	-0.19229409315399	5.17144772677035	7.92892830101040
H	-1.26436750086180	4.35986705258475	9.08785143994608
H	0.33902178556442	3.70054732505809	8.76275047441895
C	-4.19316948428417	9.64998289611272	11.88196740425694
H	-4.90141525212988	10.03395072268023	12.62508710835871
H	-4.75139083214933	9.02796755033480	11.17343309920074
H	-3.78452320442236	10.50827656805932	11.33857370529514
C	1.35108241238437	12.77591824702027	13.36879029024302
H	0.64201664433717	13.05742718776992	12.58359886882024
H	2.35835873391564	13.05623320450504	13.04086570818634
H	1.11327772609229	13.36206288758572	14.26376735231792
B	0.90335569856757	7.38630455623714	14.27032403539840
O	1.73853987493220	7.15621492355490	15.33795776122884
O	-0.42158617369412	7.11584470946919	14.53216784621564
C	0.87640059412919	6.94732572037502	16.49556170228996
C	-0.46291356034347	6.45804384858192	15.83515412717292
C	1.53439329781554	5.93856252325247	17.41978843231342
C	0.73169636613592	8.29987095896571	17.18854018370363
C	-0.48535633729384	4.95627659553280	15.56614948728269
C	-1.71882567136203	6.89256036930128	16.57100204193672
H	0.86800659306538	5.69245794811792	18.25369625750051
H	1.79323411333670	5.01896524817905	16.89116568309152
H	2.45306238760673	6.36545129688448	17.83306051255255
H	0.15865655580651	8.21361031869512	18.11706224130179
H	1.72906516406354	8.67900513183243	17.43071332602787
H	0.23725912406757	9.02869981227190	16.53776626171865
H	-0.58521281932554	4.38462858284413	16.49427865268786
H	-1.34173099964111	4.72688373859880	14.92488781573178
H	0.42385664430839	4.63250106733777	15.04955479559924
H	-1.69824164149406	6.52719477213115	17.60361822784869
H	-1.81775764332178	7.98058440218253	16.57806461509044
H	-2.60249275675242	6.47287560650410	16.08173144872267
O	1.38360794639730	7.80643544994263	13.07083606096923

Nacnac^{Mes}Zn(DMAP)Bpin (2): -3577.947517146553 E_h

Zn	-1.26553191673102	9.20671671871936	15.25186422618441
O	0.62814103947869	8.95479881441431	12.82191992819689
O	0.36528032194073	6.96907404899294	13.91345855822676
N	-1.06392003773432	9.37679795263189	17.26035161109705
N	-3.23414404414391	8.70999275545253	15.38177745620870
N	-1.52908695091188	11.29520777089302	14.80891942924737

N	-2.16731187117123	15.32451903815807	13.87917139410685
C	-3.94417620553340	8.76278782690625	16.49763279302369
C	0.27484094683242	9.36954430087198	17.74286749969442
C	-1.95655840408870	14.01361082010496	14.18365343195196
C	-0.88916569858478	11.88352962182920	13.78554778262874
H	-0.21024017985852	11.24411579905140	13.22309972612208
C	-3.78508436822343	8.11529808565273	14.21250446333112
C	-2.07306614194328	9.33398643407010	18.11815566495661
C	-2.62225873772952	13.38259550188070	15.26002643524619
H	-3.32436641605613	13.91701385445641	15.88781789729889
C	0.96190396124200	10.57013083073125	17.98277179435226
C	-3.42170439523415	9.15629630439873	17.74741536416273
H	-4.13427310476222	9.17767826726401	18.56327070485789
C	-1.06287572691330	13.20884228336104	13.44077689278275
H	-0.50613343476579	13.60418008719275	12.60032389318660
C	0.92749093711957	8.13556286555375	17.90783189838368
C	-5.39411309526736	8.33131165259412	16.46894299648225
H	-5.93710563272217	8.81330884468875	15.64963077116276
H	-5.89260034311823	8.55574714577229	17.41366491507839
H	-5.46121237817722	7.25153965165966	16.28917057075596
C	1.58355134526437	8.03202607580994	12.21378632870030
C	-4.44027254136962	8.89624037930226	13.24751893985695
C	2.28936571473082	10.51310303253106	18.40768077141137
H	2.81686115071809	11.44528859973450	18.60557961334156
C	2.95665978459078	9.30245705695284	18.58263909446783
C	-2.37849293749274	12.05155286502302	15.52227636220139
H	-2.88659594640651	11.54957684709012	16.34509156086150
C	-4.62801498089666	10.37564179980695	13.44120480757852
H	-3.70263750322077	10.91804092198689	13.21372369902822
H	-4.88977574184495	10.62660707342213	14.47351062815040
H	-5.41195195163585	10.75545499486912	12.77948052294114
C	2.25672910223635	8.12466342147698	18.32747464060057
H	2.75662789845649	7.16645827216380	18.45911827102763
C	-3.59329956680569	6.73860137821030	13.99626623571138
C	4.40052328980350	9.27151602350944	19.00753852808011
H	5.06778056102759	9.40307054960300	18.14645297948997
H	4.65638973484680	8.31750011118451	19.47794618528025
H	4.62452415983883	10.07339319295949	19.71836318544659
C	-1.78036775358060	9.42858535426770	19.59986331680805
H	-1.23584728915769	8.53650968096788	19.93179473401442
H	-2.70102361673284	9.50827558032556	20.18051609835974
H	-1.13459612917668	10.28328716634244	19.82528793196243
C	1.01824420291861	6.64545667544002	12.64990854737005
C	0.29685408016528	11.90255007379514	17.77160084144748
H	0.84133776096735	12.69313953123065	18.29619177363537
H	-0.74148686290990	11.90481546524815	18.11653792148049
H	0.27161936037104	12.15998355275500	16.70593242501789
C	0.20509405092519	6.85200925603445	17.61176184284935
H	0.79927361377384	5.99024480228407	17.92810452289015
H	0.01734794849685	6.75997120906963	16.53455978936514
H	-0.77026754797415	6.80633318455014	18.10831062515848

C	-4.90624690336926	8.28152217699090	12.08453199526003
H	-5.42408197584109	8.88818079176188	11.34295010284350
C	-3.06898833414687	16.12657773421233	14.69214581236577
H	-4.09002972229802	15.72140727711195	14.68063267801040
H	-3.10671973706630	17.14095584464822	14.29280121583255
H	-2.73323848037799	16.18485568020135	15.73735042490094
C	-1.43828710185272	15.94562152516713	12.78379448954290
H	-0.35227017840812	15.92587552602433	12.95340972673260
H	-1.74733405685107	16.98769161124066	12.69197701308686
H	-1.64654038513497	15.44660099962431	11.82762526362192
C	-2.86025101937157	5.90466533755352	15.00767228455904
H	-2.96012537451184	4.84021856635251	14.77684799241215
H	-3.22395857253546	6.07728595690756	16.02621205258793
H	-1.79234011027148	6.15558879139963	15.00229128997870
B	0.02293614467769	8.31688073157744	13.90249638014754
C	-4.07049392323907	6.16684336336941	12.81828022478338
H	-3.92390723837612	5.09986678666656	12.65705766606767
C	2.94706431838104	8.34223713327858	12.82935413523168
H	3.18149606839142	9.39666932443945	12.65454558016698
H	3.73963788651468	7.73319722110851	12.38139653934403
H	2.93458780514179	8.17239302505781	13.91030001957622
C	1.61176475786661	8.27158563620232	10.71382409595862
H	0.61181576734759	8.20996263023565	10.27937439068170
H	2.25794707262806	7.53764391007084	10.21841480393719
H	2.01331742101697	9.26903019344319	10.50891488744611
C	-4.72688105162236	6.92096580235294	11.84699739467886
C	2.06579937863317	5.57326565862179	12.89774287110351
H	2.75942553626272	5.86814565564713	13.68784724709862
H	2.63534225683707	5.37111996747664	11.98332952819973
H	1.57421570015284	4.64494545124974	13.20461443469272
C	-5.20277806972058	6.28810629909843	10.56660443856796
H	-6.07423123527803	6.81240194524293	10.16255650428035
H	-5.47509404860167	5.23908696135406	10.71820044633717
H	-4.41893669050920	6.31485290508029	9.79908627097158
C	-0.07368688426667	6.12145755048355	11.71739243415303
H	-0.56546565474143	5.26989732448132	12.19789883418369
H	0.33820099594650	5.78887568306445	10.75842225620661
H	-0.83594087427613	6.88566261595050	11.53541945118655

[Nacnac^{Mes}Zn]$_2$ (VIII): -5567.498683630937 E_h

Zn	2.64300758150056	2.28642526406573	5.81276762161217
N	2.03342380440939	1.57602673351300	7.56362145273025
N	4.28670080060830	3.10904935583277	6.56783267122283
C	2.70897416462438	1.67859941902235	8.70442870141882
C	3.94024553125163	2.34354443838709	8.82638673099562
H	4.37672084552565	2.34657191654596	9.81766159109818
C	4.66485202361652	3.03267332750375	7.83901991374136
C	2.13005561952597	1.05592540414187	9.95411131806192
H	1.17311887914037	1.52193025438396	10.21333119795595
H	2.81202195356203	1.16013572819544	10.79944608353056

H	1.91963833745642	-0.00713668493024	9.79376302073310
C	5.93912825588554	3.72353676939099	8.26615742428139
H	6.79873470277744	3.33606905547675	7.70835352787102
H	6.12149538210506	3.59199463851847	9.33386573292856
H	5.88743733837717	4.79523813248032	8.04419088187690
C	0.75100526755024	0.96229862150509	7.55151677895013
C	-0.38704078500299	1.73549717494756	7.82443894541841
C	-1.63821846008401	1.12075605779301	7.78408271562769
H	-2.52083069566166	1.71730174377987	8.00786778908926
C	-1.78686158101102	-0.22310080307772	7.44798450273359
C	-0.64113733292457	-0.95868030911241	7.14362438650270
H	-0.73785736205911	-2.00749955953545	6.86713704609573
C	0.62950127763432	-0.38746599659674	7.18587633390518
C	-0.25485132269176	3.20278707600893	8.11752645100502
H	-1.22051172590050	3.63062156785431	8.39908847567480
H	0.09825004418182	3.73872510907657	7.22596875051890
H	0.46707749446374	3.40429402400823	8.91620906318229
C	-3.14948891510594	-0.85691189132425	7.36752407232035
H	-3.89436595413214	-0.26926000674546	7.91182246896004
H	-3.14394393134557	-1.86978167461516	7.78300024180326
H	-3.48583999944737	-0.93573327141008	6.32580827489071
C	1.85454683375523	-1.18809343441223	6.84517964143024
H	1.58773429990710	-2.20914908924743	6.55984669690580
H	2.55720814287599	-1.23545396360887	7.68527318317116
H	2.40245133375378	-0.72762517537680	6.01261075188564
C	5.07051709151048	3.83672464025954	5.63152583517302
C	6.08758290536050	3.18478903654028	4.92020566239216
C	6.82143780952144	3.91433845158846	3.98454780804288
H	7.61973243034716	3.41275767528615	3.44053328912960
C	6.54576680802702	5.25242487435780	3.71451056000005
C	5.50228823294939	5.86566937881855	4.40910224417845
H	5.26293241076176	6.90778421186094	4.20260075649991
C	4.75583685640362	5.17955378902641	5.36518338834799
C	6.35029141725758	1.72219625069917	5.13802831239190
H	7.24596348394262	1.40450513283513	4.59798753797512
H	5.50836280712801	1.12399732639511	4.76342464050072
H	6.46978725572009	1.47468014250371	6.19814366725302
C	7.31775135510692	6.00914464309914	2.66744193675499
H	7.59263477638493	7.00940240262806	3.01820629783925
H	6.72000924739296	6.13832210178552	1.75625376090953
H	8.23495025156669	5.48153785435853	2.39056914745999
C	3.62800746699279	5.85076419398473	6.09775383657129
H	3.50236086824563	6.88235720884236	5.75808970048299
H	3.79337156319259	5.86235855769464	7.18149752777189
H	2.68096260805946	5.31844036658531	5.93833657601635
Zn	1.76519431368122	2.28717101252798	3.64876826035315
N	2.37419994823698	1.57697205515855	1.89769353832478
N	0.12195156070401	3.11149821054290	2.89387480884532
C	1.69845920707474	1.68004918179999	0.75700890738855
C	0.46776836747360	2.34606267282164	0.63521243888033
H	0.03125820876965	2.34958538578776	-0.35604494010298

C	-0.25615547022863	3.03580912466208	1.62265635102767
C	2.27650718580546	1.05668123818491	-0.49274471911502
H	3.23408979123103	1.52129220030094	-0.75203772155826
H	1.59461827745930	1.16181391467458	-1.33802682525712
H	2.48540450566906	-0.00668434541298	-0.33238382402917
C	-1.52957629491427	3.72825783669441	1.19551818686079
H	-2.38974298991020	3.34160940515699	1.75302390986627
H	-1.71189882326501	3.59728915360086	0.12773193460580
H	-1.47666646468820	4.79982062743029	1.41786684686409
C	3.65625364788277	0.96245644419438	1.90924235331614
C	4.79459870257808	1.73486786198635	1.63527524814513
C	6.04539385300444	1.11931817643541	1.67473617991175
H	6.92823077741457	1.71523191555917	1.45015222962222
C	6.19343713997782	-0.22459081786530	2.01094875744069
C	5.04748720956212	-0.95934989190514	2.31638408904221
H	5.14372285569336	-2.00818130014993	2.59299672238013
C	3.77717505802639	-0.38730862008662	2.27504019477214
C	4.66307656526918	3.20215387160735	1.34183885593770
H	5.62900318996933	3.62960404023356	1.06061001873421
H	4.30967927599896	3.73839405708088	2.23307903765257
H	3.94160829613819	3.40372677751863	0.54274170800459
C	7.55572779113322	-0.85923974812023	2.09044541168041
H	8.30072892630636	-0.27169232323390	1.54620969359847
H	7.54943968401241	-1.87184346622382	1.67431898571697
H	7.89244015227471	-0.93891672951081	3.13197652275406
C	2.55188350959424	-1.18717376905265	2.61663506669449
H	2.81832466647753	-2.20821209833961	2.90237701231767
H	1.84892296471288	-1.23463878239003	1.77679202913092
H	2.00446398485892	-0.72604377074607	3.44915345509268
C	-0.66120627518957	3.83974171652599	3.83025741403968
C	-1.67949371551395	3.18880009318333	4.54087983227117
C	-2.41306271538729	3.91897391731733	5.47618515071468
H	-3.21235783860178	3.41829461948730	6.01957880829103
C	-2.13603933251655	5.25677647728553	5.74662159029350
C	-1.09145546809394	5.86890509546024	5.05284277279724
H	-0.85107904137040	6.91074411456807	5.25950298467141
C	-0.34510146083453	5.18206404849751	4.09711242413416
C	-1.94392672849585	1.72663241022340	4.32225526859590
H	-2.83938110057453	1.40947523831211	4.86296747444796
H	-1.10222328380920	1.12728057142489	4.69547138054394
H	-2.06484258679408	1.48004574739432	3.26207077699211
C	-2.90765648713404	6.01381224290894	6.79371325097285
H	-3.16880104868542	7.01971075119276	6.44878988823193
H	-2.31553883347948	6.12925331110818	7.71045864240975
H	-3.83262686725161	5.49419796512397	7.05969781480762
C	0.78375763006949	5.85225243689384	3.36518915024743
H	0.91061334735144	6.88350402113528	3.70544332986449
H	0.61865639979668	5.86465403012965	2.28140964011874
H	1.73011333753537	5.31867749931471	3.52452875483176

B_2pin_2: -823.230375444391 E_h			
B	2.99647900353390	7.63231442239154	4.06880138345847
O	1.71731978187177	7.17740910901088	3.85202156783805
O	3.59569910721975	8.10143587688927	2.92390221534774
C	1.50439144922485	7.14191401881129	2.40628823254165
C	2.56648832135963	8.16966958057668	1.88799062955685
C	0.05974267529106	7.51005098666192	2.11445723968233
C	1.78790264588254	5.70905948314124	1.96121330065336
C	2.05588252001086	9.60860061230225	1.88087759079686
C	3.19563902120082	7.82407300652325	0.54909582098991
H	-0.10860356145079	7.57132546294021	1.03361458915338
H	-0.21099139528627	8.46551595705514	2.56770262587917
H	-0.60255900134467	6.73985554225316	2.52050423568369
H	1.58783061136296	5.57601225250869	0.89322545029313
H	1.13905123461885	5.03116748285422	2.52307290965889
H	2.82701503234689	5.43084605015237	2.16206963559285
H	1.32180638018239	9.76912273747494	1.08480944191630
H	2.90229460511230	10.28082822999508	1.71402205398941
H	1.59849279220583	9.86867531643926	2.84029917313629
H	2.43239906454102	7.79329890066217	-0.23626624699273
H	3.70648996351157	6.85986035997933	0.58133419270878
H	3.92930546761676	8.59039091538860	0.28231339114695
B	3.75515838328411	7.61768442312303	5.59104708386602
O	5.03431617004404	8.07259389668102	5.80782679451489
O	3.15594054949252	7.14855940099526	6.73594597200860
C	5.24724503085427	8.10808816453954	7.25356007271885
C	4.18515214413024	7.08032812415630	7.77185693554083
C	6.69189533013489	7.73995625594299	7.54539010757866
C	4.96372884619389	9.54094118390420	7.69863672682009
C	4.69576323521996	5.64139896094510	7.77896779675303
C	3.55600097439349	7.42592069916558	9.11075254458754
H	6.86024223165445	7.67868135271049	8.62623262880590
H	6.96263276493435	6.78449273681250	7.09214367295325
H	7.35419400983545	8.51015454127284	7.13934360649596
H	5.16380084814770	9.67398797201758	8.76662464383819
H	5.61257759787193	10.21883610231875	7.13677757157077
H	3.92461539722164	9.81915118868052	7.49778115063802
H	5.42984059750986	5.48087858734135	8.57503518155547
H	3.84935375376542	4.96916803046555	7.94582319691846
H	5.15315313922848	5.38132715442136	6.81954550450191
H	4.31924131128947	7.45669663055966	9.89611416807490
H	3.04514646309679	8.39013150518658	9.07851566701614
H	2.82233750268491	6.65959978474801	9.37753454021103

8. References for SI

S1 S. Schulz, T. Eisenmann, U. Westphal, S. Schmidt, U. Flörke, *Zeitschrift für anorganische und allgemeine Chemie* **2009**, *635*, 216-220.

S2 J. Prust, A. Stasch, W. Zheng, H. W. Roesky, E. Alexopoulos, I. Usón, D. Böhler, T. Schuchardt, *Organometallics* **2001**, *20*, 3825-3828.

S3 Y. K. Loh, L. Ying, M. Á. Fuentes, D. C. H. Do, S. Aldridge, *Angewandte Chemie International Edition* **2019**, *58*, 4847-4851.

S4 J. Cosier, A. M. Glazer, *Journal of Applied Crystallography* **1986**, *19*, 105-107.

S5 A. Technologies, **2011**.

S6 G. Sheldrick, *Acta Crystallographica Section A* **2015**, *71*, 3-8.

S7 G. Sheldrick, *Acta Crystallographica Section C* **2015**, *71*, 3-8.

S8 O. V. Dolomanov, L. J. Bourhis, R. J. Gildea, J. A. K. Howard, H. Puschmann, *Journal of Applied Crystallography* **2009**, *42*, 339-341.

S9 J. Bykowski, J. Sinclair, J. Trach, M. J. Ferguson, E. Rivard, *Dalton Transactions* **2023**, *52*, 1602-1607.

S10 G. Bendt, S. Schulz, J. Spielmann, S. Schmidt, D. Bläser, C. Wölper, *European Journal of Inorganic Chemistry* **2012**, *2012*, 3725-3731.

S11 F. Neese, *WIREs Computational Molecular Science* **2012**, *2*, 73-78.

S12 F. Neese, *WIREs Computational Molecular Science* **2022**, *12*, e1606.

S13 S. Grimme, A. Hansen, S. Ehlert, J.-M. Mewes, *The Journal of Chemical Physics* **2021**, *154*, 064103.

S14 J.-D. Chai, M. Head-Gordon, *Physical Chemistry Chemical Physics* **2008**, *10*, 6615-6620.

S15 E. Caldeweyher, C. Bannwarth, S. Grimme, *The Journal of Chemical Physics* **2017**, *147*, 034112.

S16 F. Weigend, R. Ahlrichs, *Physical Chemistry Chemical Physics* **2005**, *7*, 3297-3305.

S17 J. K. B. E. D. Glendening, A. E. Reed, J. E. Carpenter, J. A. Bohmann, C. M. Morales, P. Karafiloglou, C. R. Landis, F. Weinhold, **2018**.

S18 E. D. Glendening, C. R. Landis, F. Weinhold, *Journal of Computational Chemistry* **2019**, *40*, 2234-2241.

S19 T. Lu, F. Chen, *Journal of Computational Chemistry* **2012**, *33*, 580-592.
